# Supplementary material for: Triterpene Glycosides from the Far Eastern Sea Cucumber Psolus chitonoides: Chemical Structures and Cytotoxicities of Chitonoidosides E1, F, G, and H
Source: Mar Drugs. 2021 Dec 7;19(12):696. doi: 10.3390/md19120696 (PMC8708177; doi:10.3390/md19120696)
Supplement: Supplementary file 1 [file marinedrugs-19-00696-s001.zip › marinedrugs-1488465-supplementary.pdf]

Supplementary data content page

Title: **Triterpene Glycosides from the Far Eastern Sea Cucumber *Psolus chitonoides*: Chemical Structures and Cytotoxicities of Chitonoidosides E_1, F, G, and H.**

Authors: Alexandra S. Silchenko[1], Anatoly I. Kalinovsky[1], Sergey A. Avilov[1], Pelageya V. Andrijaschenko[1], Roman S. Popov[1], Ekaterina A. Chingizova[1], Vladimir I. Kalinin[1] and Dmitrenok P.S. [1]

Address: [1]*G.B. Elyakov Pacific Institute of Bioorganic Chemistry, Far Eastern Branch of Russian Academy of Sciences, Pr. 100-let Vladivostoka 159, 690022 Vladivostok, Russia*

Correspondence kalininv@piboc.dvo.ru; Tel.: +7-423-231-1168; paveldmt@piboc.dvo.ru

Content:

Figure S1. The ^{13}C NMR (125.67 MHz) spectrum of chitonoidoside E_1 (**1**) in C_5D_5N/D_2O (4/1)

Figure S2. The ^{1}H NMR (500.12 MHz) spectrum of chitonoidoside E_1 (**1**) in C_5D_5N/D_2O (4/1)

Figure S3. The COSY (500.12 MHz) spectrum of chitonoidoside E_1 (**1**) in C_5D_5N/D_2O (4/1)

Figure S4. The HSQC (500.12 MHz) spectrum of chitonoidoside E_1 (**1**) in C_5D_5N/D_2O (4/1)

Figure S5. The ROESY (500.12 MHz) spectrum of chitonoidoside E_1 (**1**) in C_5D_5N/D_2O (4/1)

Figure S6. The HMBC (500.12 MHz) spectrum of chitonoidoside E_1 (**1**) in C_5D_5N/D_2O (4/1)

Figure S7. 1 D TOCSY (500.12 MHz) spectra of Xyl1, Qui2, Xyl3, MeGlc4, Glc5 and MeXyl6 of chitonoidoside E_1 (**1**) in C_5D_5N/D_2O (4/1)

Figure S8. HR-ESI-MS and ESI-MS/MS spectra of chitonoidoside E_1 (**1**)

Table S1. ^{13}C and ^{1}H NMR chemical shifts, HMBC and ROESY correlations of aglycone moiety of chitonoidoside E_1 (**1**)

Figure S9. The ^{13}C NMR (125.67 MHz) spectrum of chitonoidoside F (**2**) in C_5D_5N/D_2O (4/1)

Figure S10. The ^{1}H NMR (500.12 MHz) spectrum of chitonoidoside F (**2**) in C_5D_5N/D_2O (4/1)

Figure S11. The COSY (500.12 MHz) spectrum of chitonoidoside F (**2**) in C_5D_5N/D_2O (4/1)

Figure S12. The HSQC (500.12 MHz) spectrum of chitonoidoside F (**2**) in C_5D_5N/D_2O (4/1)

Figure S13. The HMBC (500.12 MHz) spectrum of chitonoidoside F (**2**) in C_5D_5N/D_2O (4/1)

Figure S14. The ROESY (500.12 MHz) spectrum of chitonoidoside F (**2**) in C_5D_5N/D_2O (4/1)

Figure S15. 1D TOCSY (500.12 MHz) spectra of Xyl1, Qui2, Glc3 and MeGlc4 of chitonoidoside F (2) in C_5D_5N/D_2O (4/1).

Figure S16. HR-ESI-MS and ESI-MS/MS spectra of chitonoidoside F (**2**)

Table S2. ^{13}C and ^{1}H NMR chemical shifts, HMBC and ROESY correlations of the aglycone part of chitonoidoside F (**2**)

Figure S17. The ^{13}C NMR (125.67 MHz) spectrum of chitonoidoside G (**3**) in C_5D_5N/D_2O (4/1)

Figure S18. The ^{1}H NMR (500.12 MHz) spectrum of chitonoidoside G (**3**) in C_5D_5N/D_2O (4/1)

Figure S19. The COSY (500.12 MHz) spectrum of chitonoidoside G (**3**) in C_5D_5N/D_2O (4/1)

Figure S20. The HSQC (500.12 MHz) spectrum of chitonoidoside G (**3**) in C_5D_5N/D_2O (4/1)

Figure S21. The HMBC (500.12 MHz) spectrum of chitonoidoside G (**3**) in C_5D_5N/D_2O (4/1)

Figure S22. The ROESY (500.12 MHz) spectrum of chitonoidoside G (**3**) in C_5D_5N/D_2O (4/1)

Figure S23. 1 D TOCSY (500.12 MHz) spectra of Xyl1, Qui2, Xyl3, MeGlc4, Glc5 and MeGlc6 of chitonoidoside G (**3**) in C_5D_5N/D_2O (4/1)

Figure S24. HR-ESI-MS and ESI-MS/MS spectra of chitonoidoside G (**3**)

Figure S25. The ^{13}C NMR (125.67 MHz) spectrum of chitonoidoside H (**4**) in C_5D_5N/D_2O (4/1)

Figure S26. The ^{1}H NMR (500.12 MHz) spectrum of chitonoidoside H (**4**) in C_5D_5N/D_2O (4/1)

Figure S27. The COSY (500.12 MHz) spectrum of chitonoidoside H (**4**) in C_5D_5N/D_2O (4/1)

Figure S28. The HSQC (500.12 MHz) spectrum of chitonoidoside H (**4**) in C_5D_5N/D_2O (4/1)

Figure S29. The ROESY (500.12 MHz) spectrum of chitonoidoside H (**4**) in C_5D_5N/D_2O (4/1)

Figure S30. The HMBC (500.12 MHz) spectrum of chitonoidoside H (**4**) in C_5D_5N/D_2O (4/1)

Figure S31. 1D TOCSY (500.12 MHz) spectra of Xyl1, Qui2, Xyl3, Glc4 and MeGlc5 of chitonoidoside H (**4**) in C_5D_5N/D_2O (4/1)

Figure S32. 2D TOCSY (500.12 MHz) spectrum of chitonoidoside H (**4**) in C_5D_5N/D_2O (4/1)

Figure S33. HR-ESI-MS and ESI-MS/MS spectra of chitonoidoside H (**4**)

Table S3. ^{13}C and ^{1}H NMR chemical shifts, HMBC and ROESY correlations of the aglycone part of chitonoidoside H (**4**)

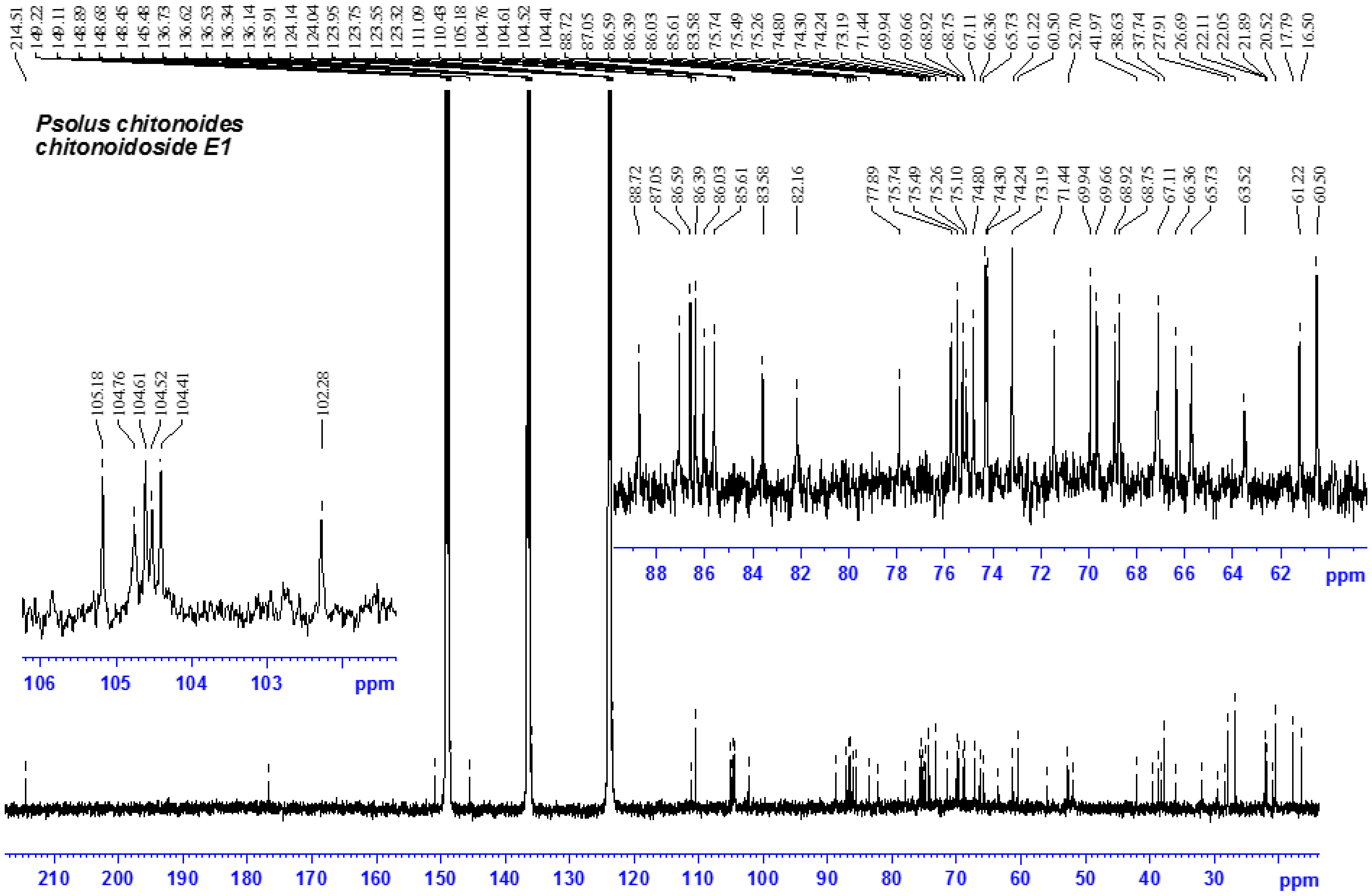

Figure S1. The ^{13}C NMR (125.67 MHz) spectrum of chitonoidoside E_1 (**1**) in C_5D_5N/D_2O (4/1)

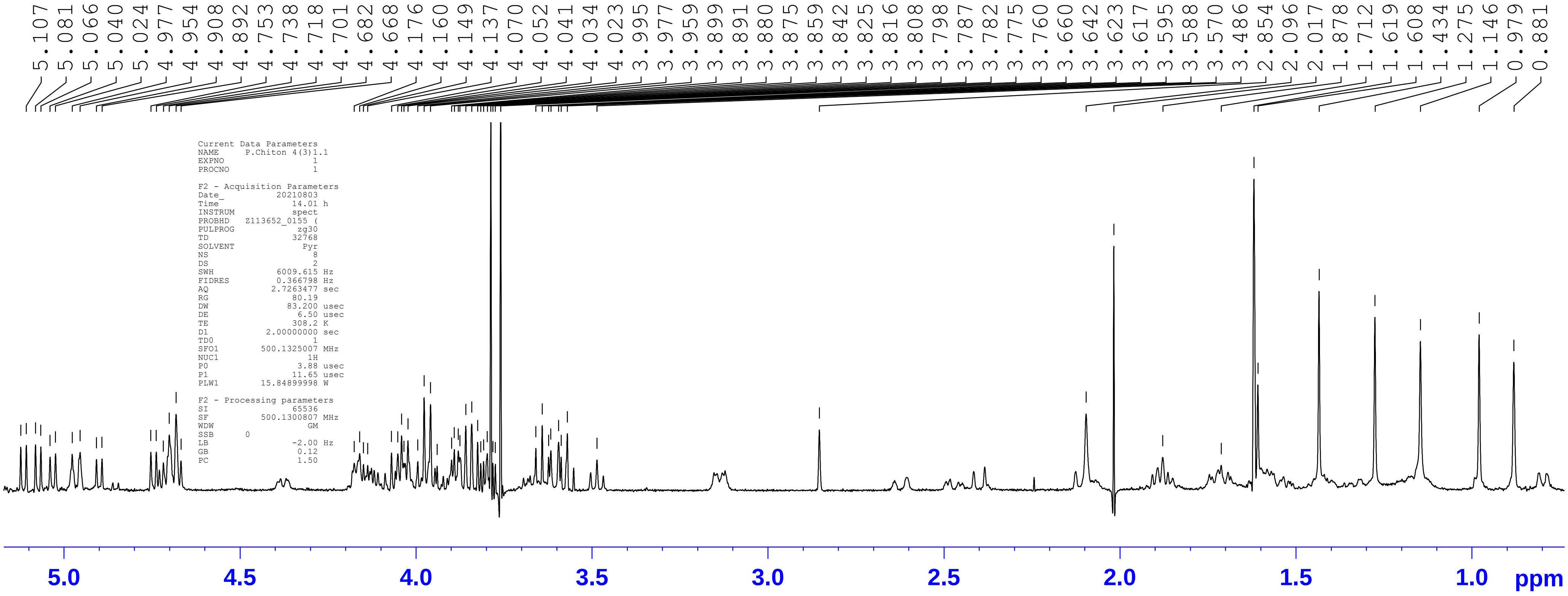

Figure S2. The ^{1}H NMR (500.12 MHz) spectrum of chitonoidoside E$_1$ (**1**) in C_5D_5N/D_2O (4/1)

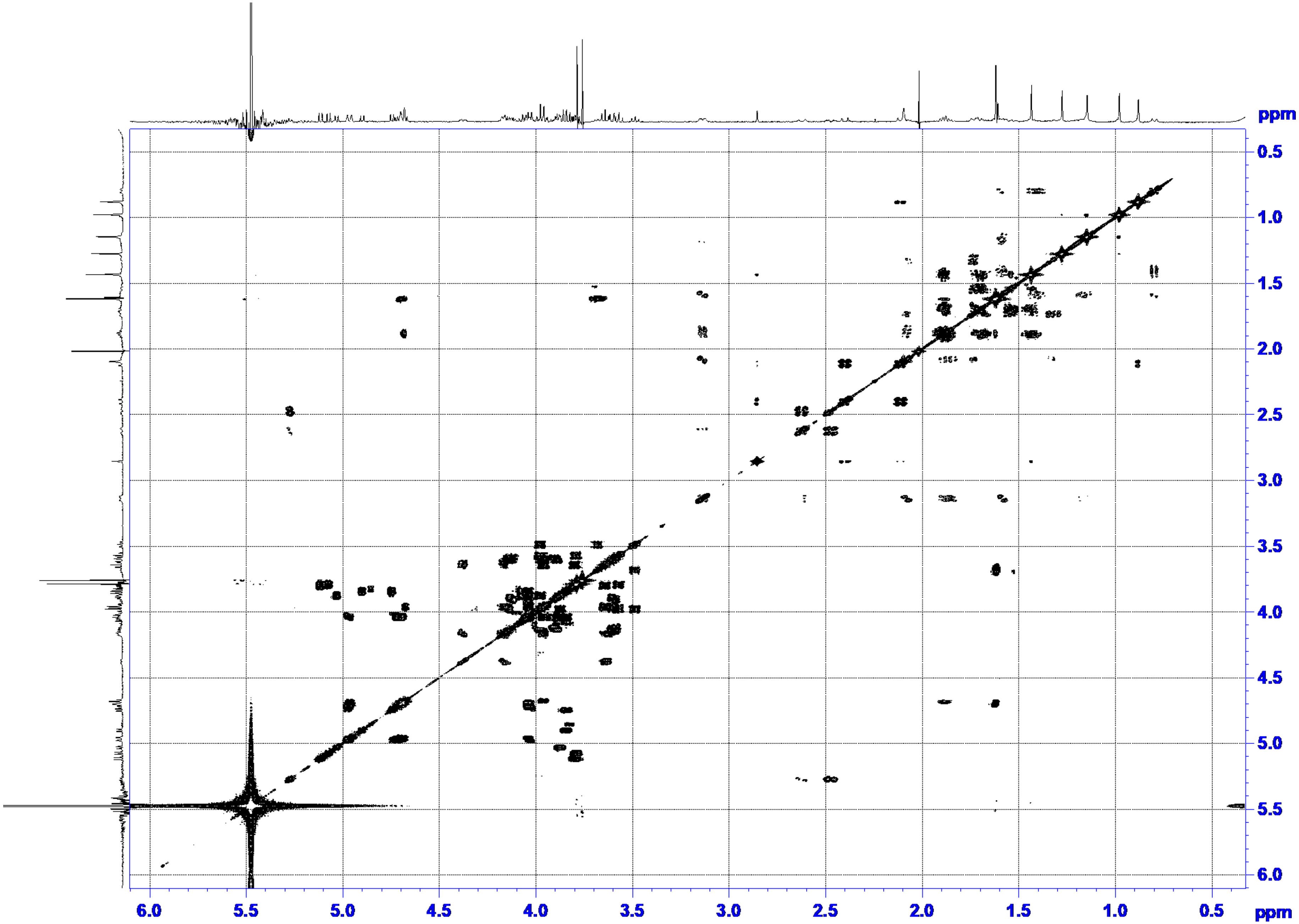

Figure S3. The COSY (500.12 MHz) spectrum of chitonoidoside E_1 (**1**) in C_5D_5N/D_2O (4/1)

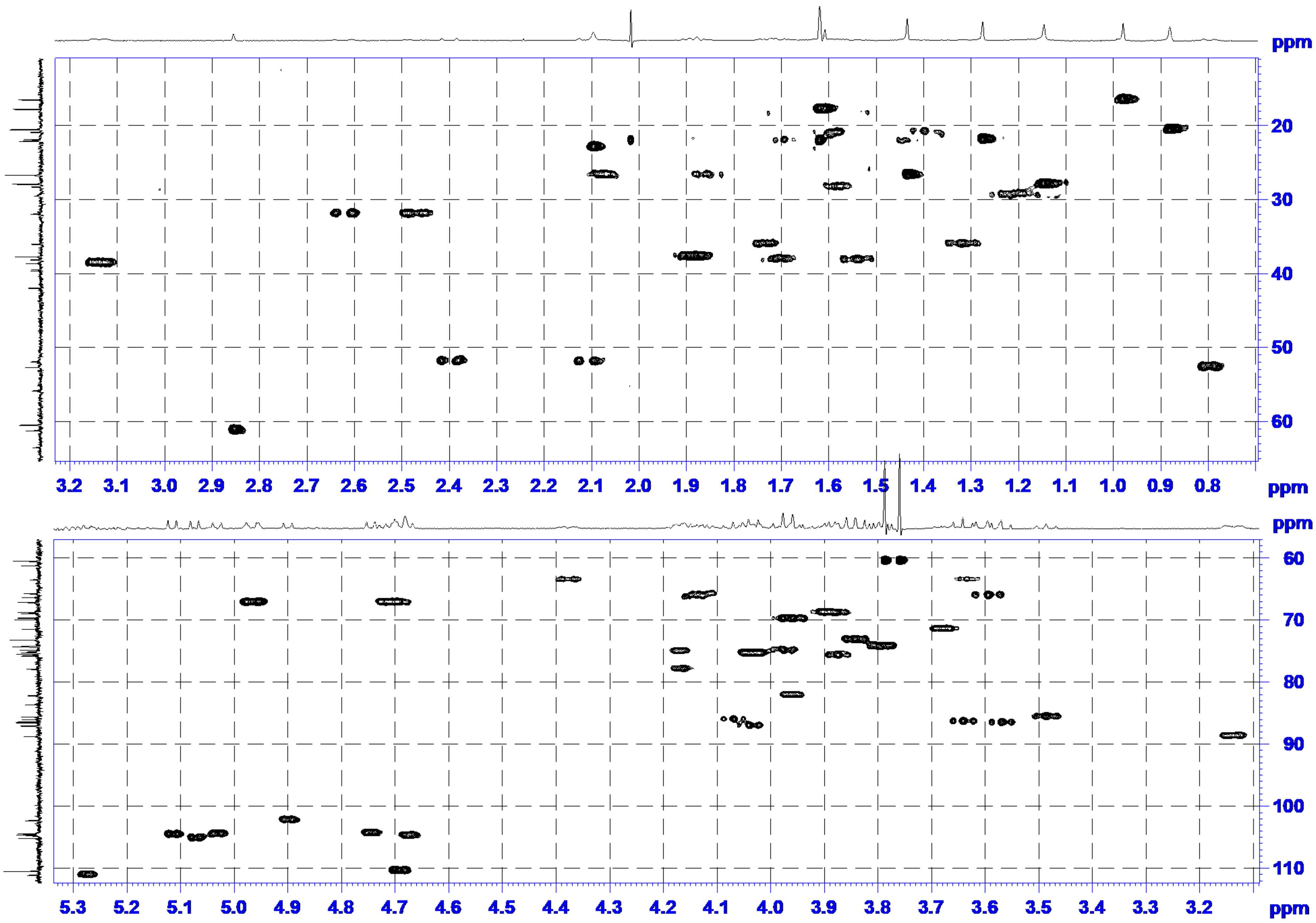

Figure S4. The HSQC (500.12 MHz) spectrum of chitonoidoside E_1 (**1**) in C_5D_5N/D_2O (4/1)

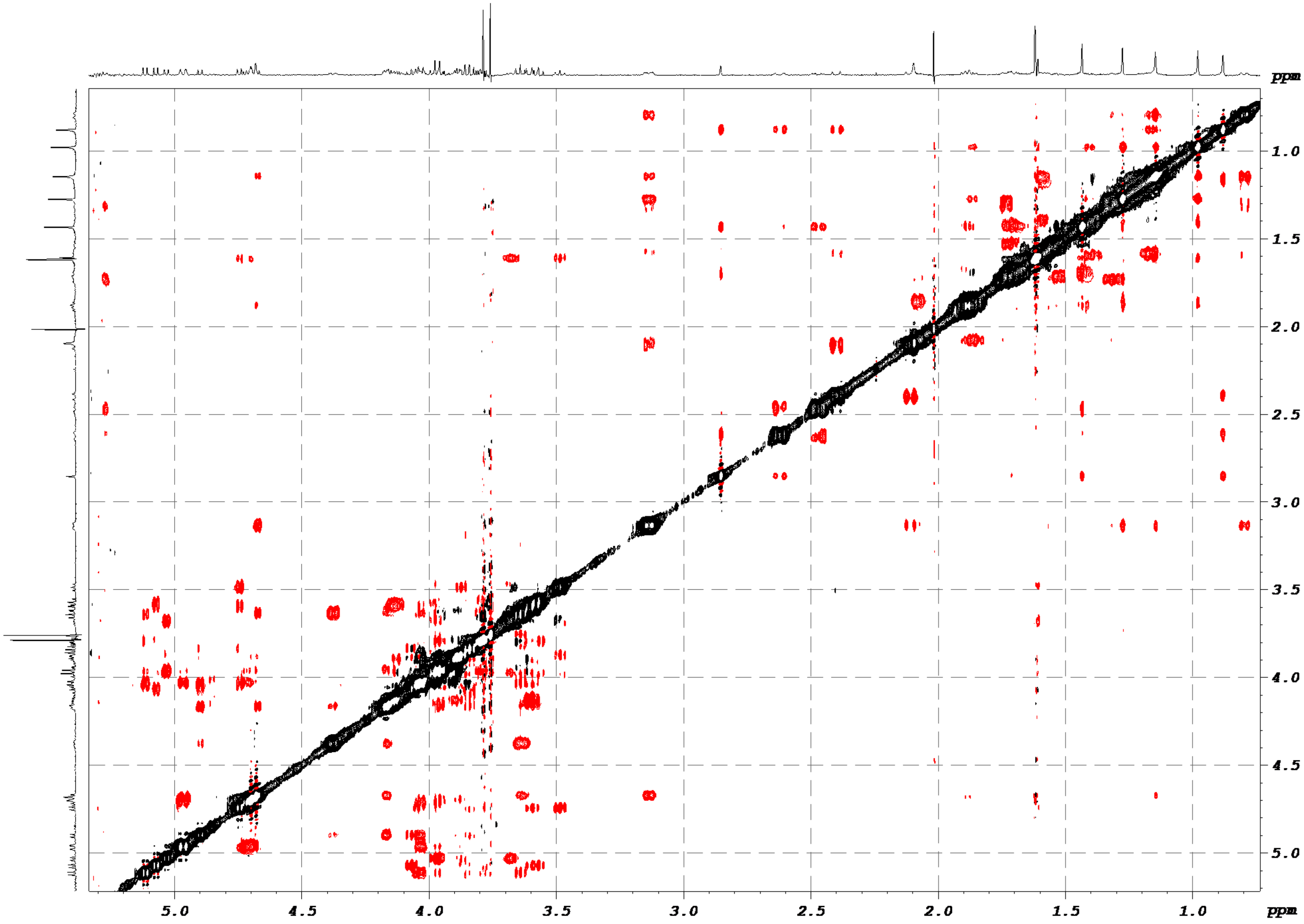

Figure S5. The ROESY (500.12 MHz) spectrum of chitonoidoside E_1 (**1**) in C_5D_5N/D_2O (4/1)

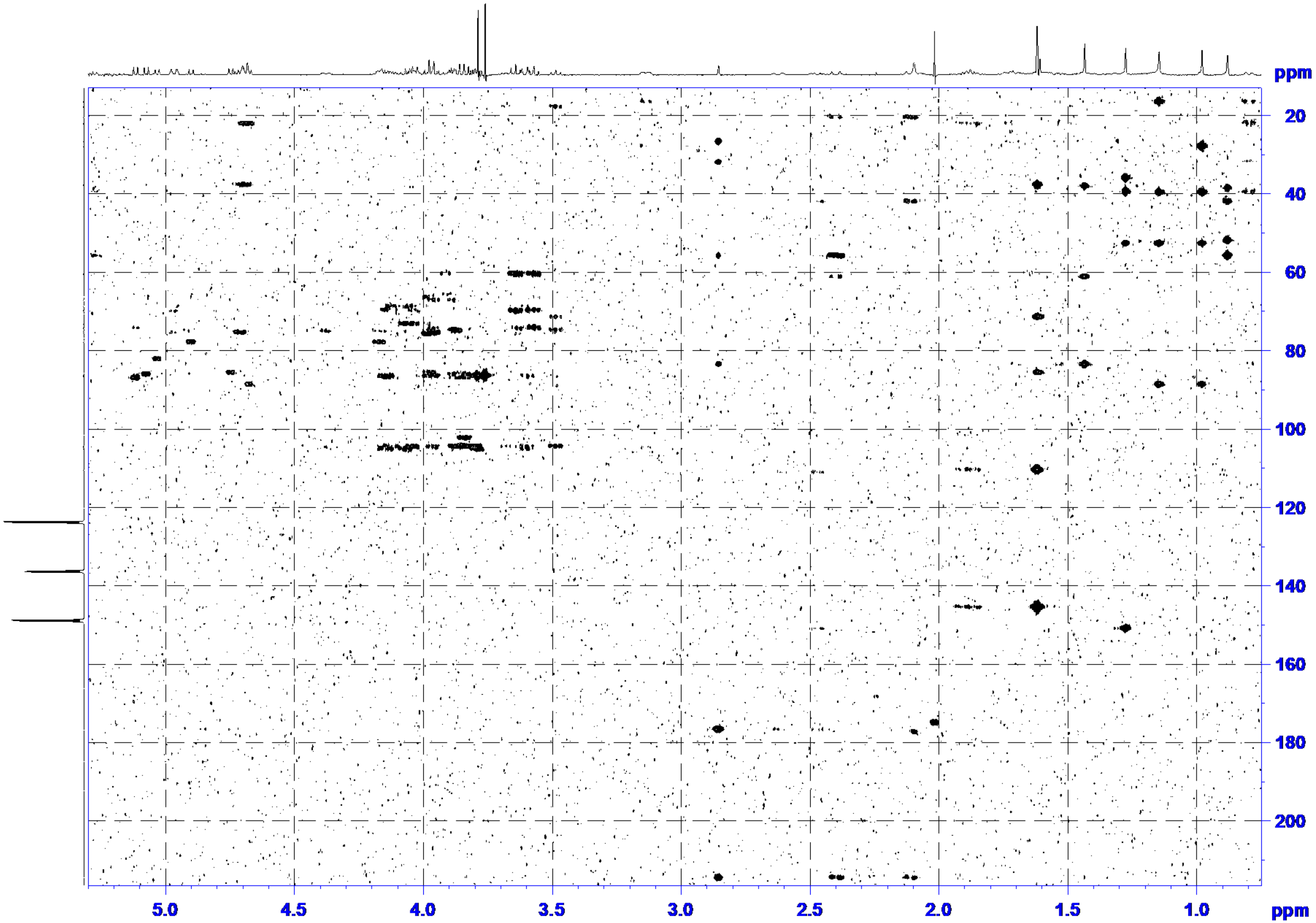

Figure S6. The HMBC (500.12 MHz) spectrum of chitonoidoside E_1 (**1**) in C_5D_5N/D_2O (4/1)

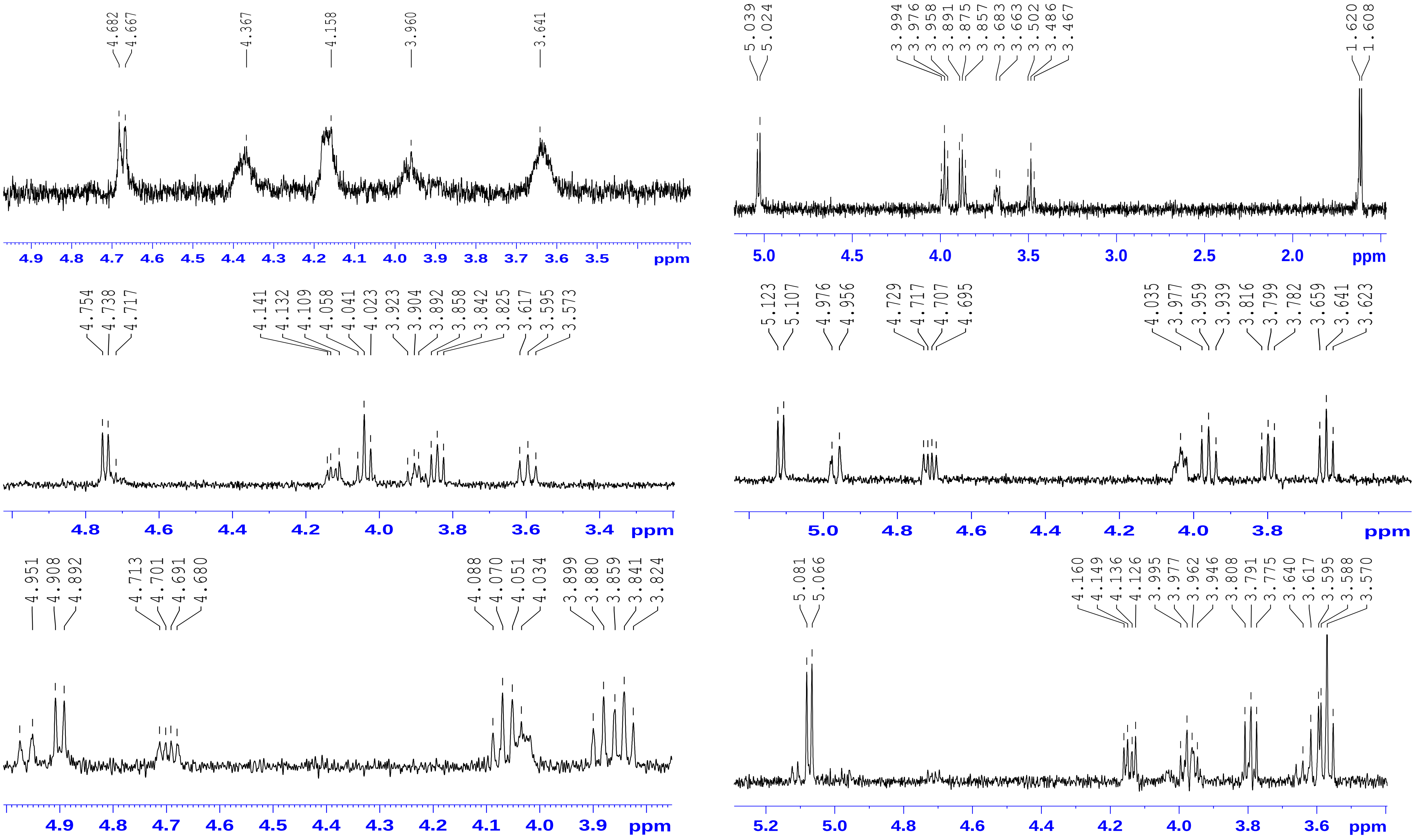

Figure S7. 1 D TOCSY (500.12 MHz) spectra of Xyl1, Qui2, Xyl3, MeGlc4, Glc5 and MeXyl6 of chitonoidoside E_1 (**1**) in C_5D_5N/D_2O (4/1)

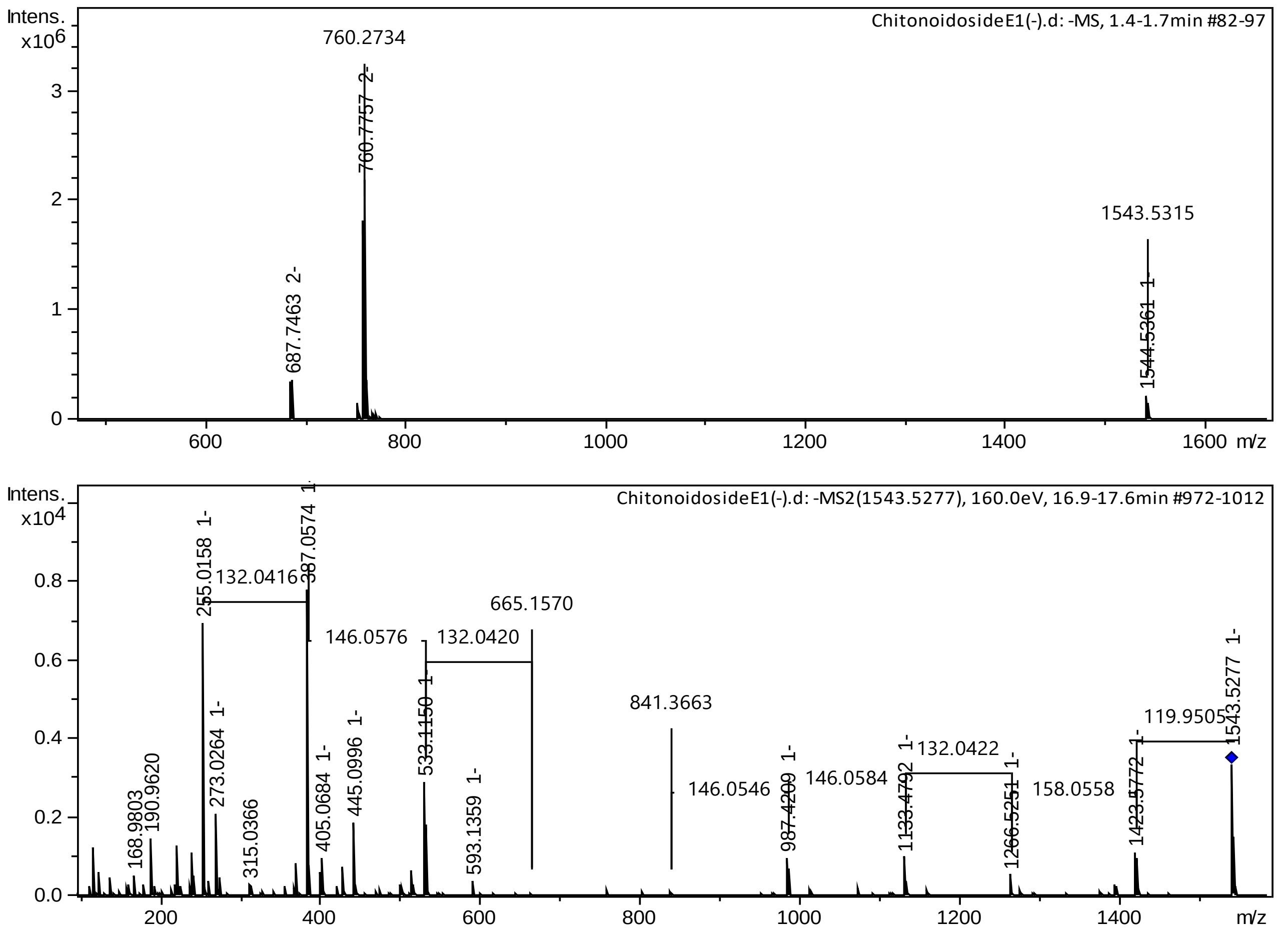

Figure S8. HR-ESI-MS (–) and ESI-MS/MS (–) spectra of chitonoidoside E1 (**1**)

Table S1. ^{13}C and ^{1}H NMR chemical shifts and HMBC and ROESY correlations of aglycone moiety of chitonoidoside E_1 (**1**).

Position	δ_C mult.[a]	δ_H mult. (*J* in Hz)[b]	HMBC	ROESY
1	36.0 CH_2	1.73 m		H-11, H-19
		1.32 m		H-3, H-5, H-11
2	27.0 CH_2	2.08 m		
		1.86 m		H-19, H-30
3	88.7 CH	3.14 dd (4.1; 11.9)	C: 4, 30, 31, C:1 Xyl1	H-1, H-5, H-31, H1-Xyl1
4	39.5 C			
5	52.7 CH	0.79 brd (11.6)	C: 6, 10, 19, 30	H-1, H-3, H-31
6	20.9 CH_2	1.59 m		H-31
		1.40 m		H-19, H-30
7	28.3 CH_2	1.58 m		
		1.12 m		H-32
8	38.6 CH	3.14 m		H-6, H-19
9	151.0 C			
10	39.6 C			
11	111.1 CH	5.28 m	C: 8, 10, 12, 13	H-1
12	32.0 CH_2	2.62 d (16.6)	C: 9, 11, 13, 18	H-17, H-32
		2.47 dd (5.0; 16.6)	C: 9, 11, 13, 14, 18	
13	55.8 C			
14	42.0 C			
15	51.9 CH_2	2.40 d (15.7)	C: 13, 16, 17, 32	H-7, H-32
		2.11 d (15.7)	C: 8, 14, 16, 32	
16	214.5 C			
17	61.2 CH	2.86 s	C: 12, 13, 16, 18, 20, 21	H-21, H-32
18	177.7 C			
19	21.9 CH_3	1.28 s	C: 1, 5, 9, 10	H-1, H-2, H-8, H-30
20	83.6 C			
21	26.7 CH_3	1.44 s	C: 17, 20, 22	H-12, H-17
22	38.1 CH_2	1.70 m	C: 20, 21, 23	
		1.54 m	C: 17, 20, 21, 23	
23	22.0 CH_2	1.71 m		
		1.45 m		
24	37.7 CH_2	1.88 m	C: 22, 25, 26, 27	H-21
25	145.5 C			
26	110.4 CH_2	4.70 brs	C: 24, 27	
		4.68 brs	C: 24, 27	
27	22.1 CH_3	1.62 s	C: 24, 25, 26	
30	16.5 CH_3	0.98 s	C: 3, 4, 5, 31	H-31
31	27.9 CH_3	1.15 s	C: 3, 4, 5, 30	H-3, H-5, H-30
32	20.5 CH_3	0.88 s	C: 8, 13, 14, 15	H-7, H-12, H-17

[a] Recorded at 125.67 MHz in C_5D_5N. [b] Recorded at 500.12 MHz in C_5D_5N.

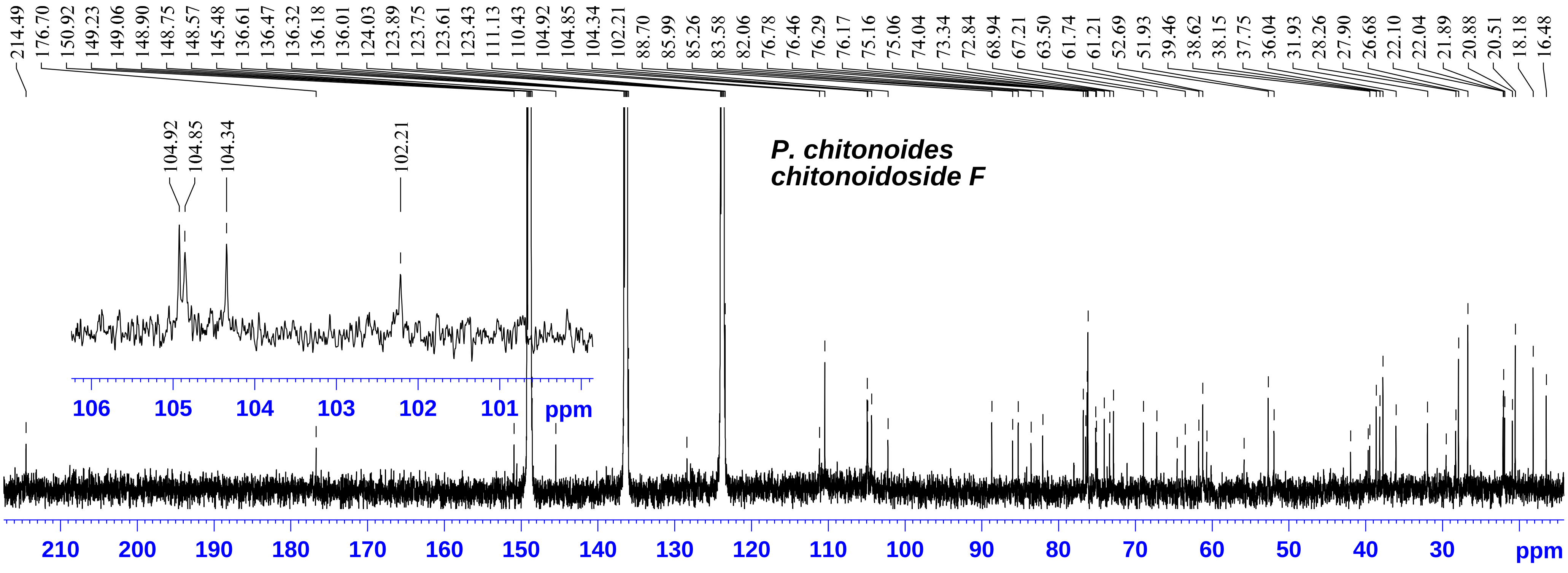

Figure S9. The ^{13}C NMR (125.67 MHz) spectrum of chitonoidoside F (**2**) in C_5D_5N/D_2O (4/1)

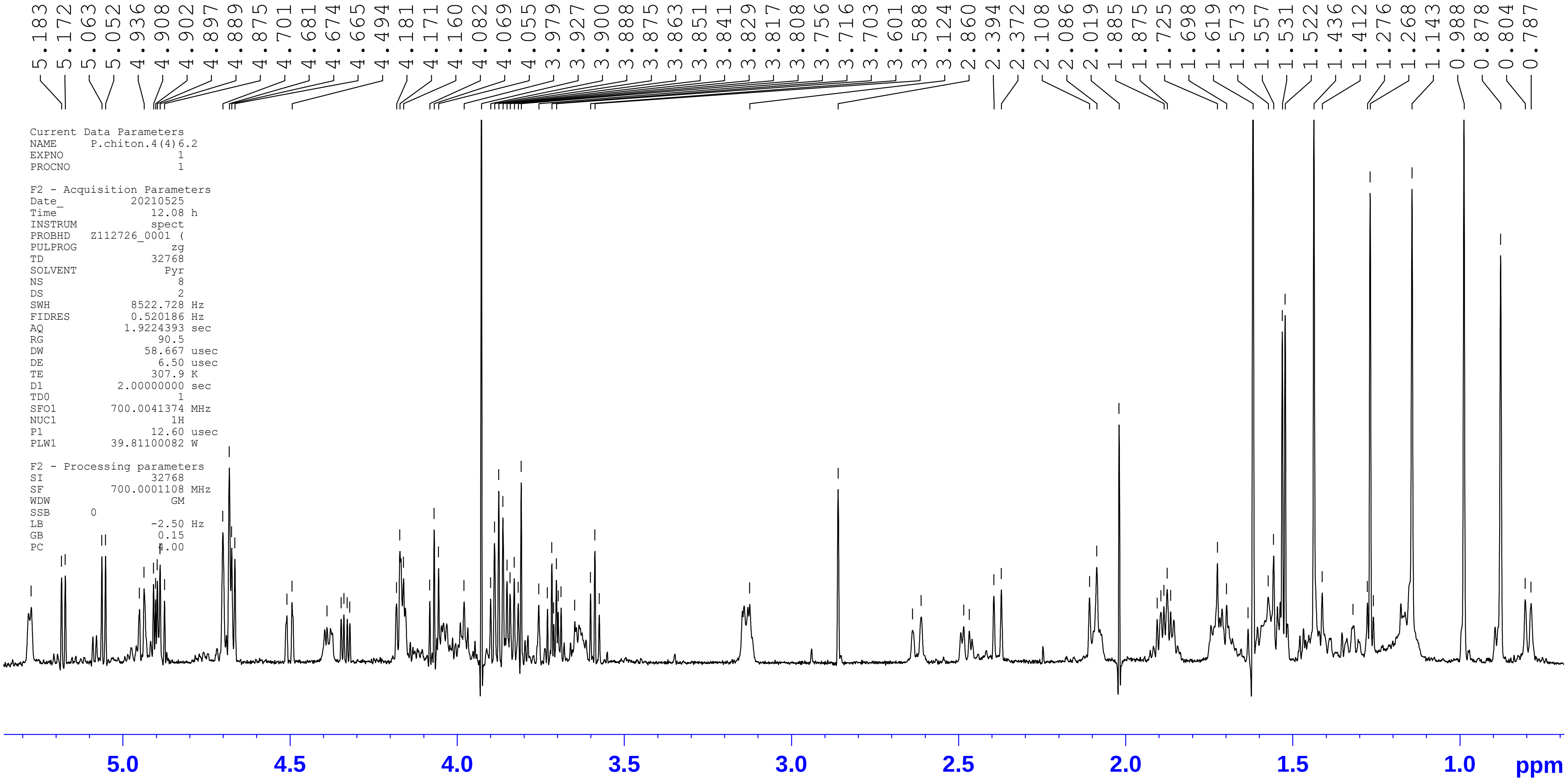

Figure S10. The ^{1}H NMR (500.12 MHz) spectrum of chitonoidoside F (**2**) in C_5D_5N/D_2O (4/1).

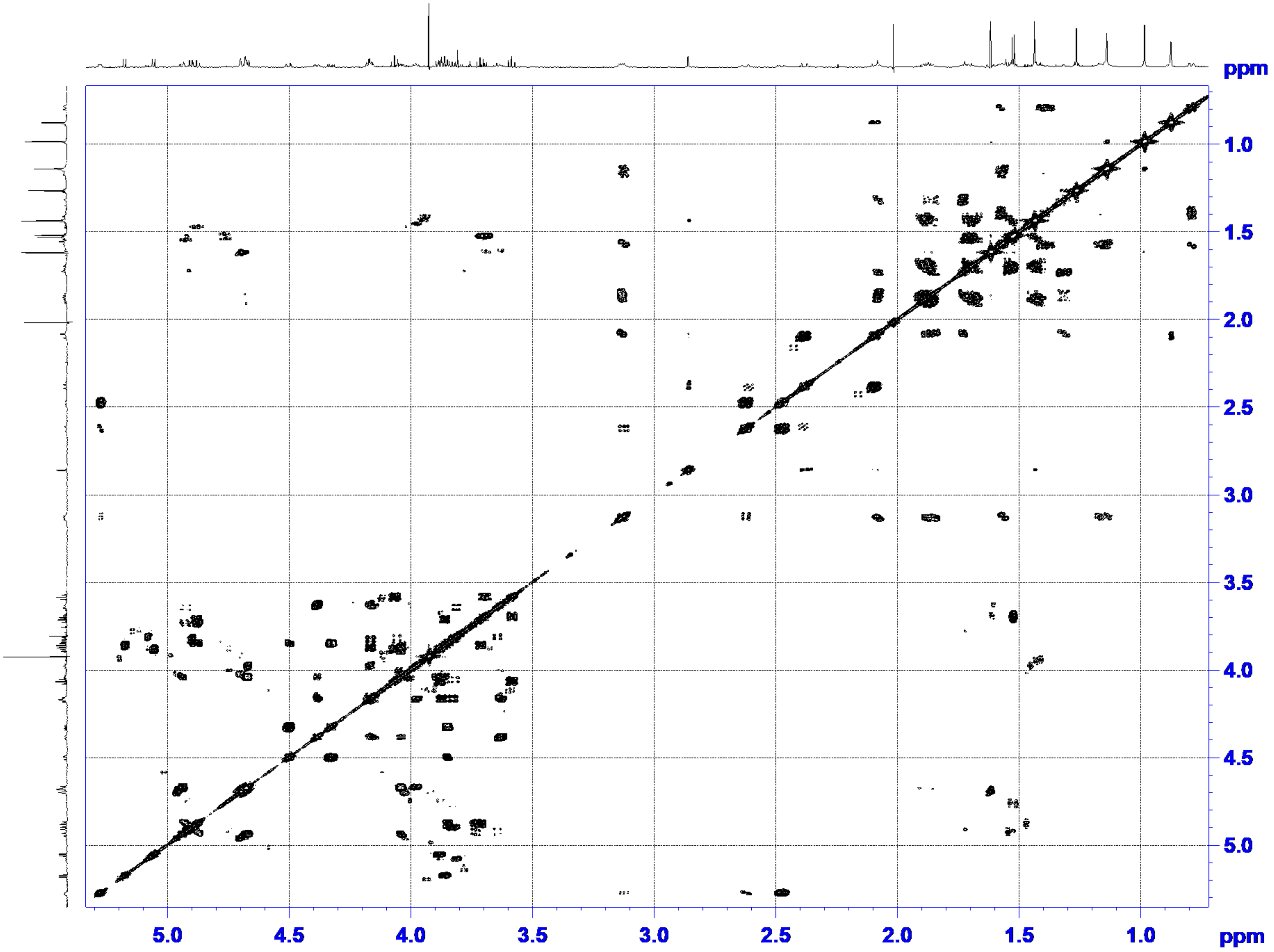

Figure S11. The COSY (500.12 MHz) spectrum of chitonoidoside F (**2**) in C_5D_5N/D_2O (4/1).

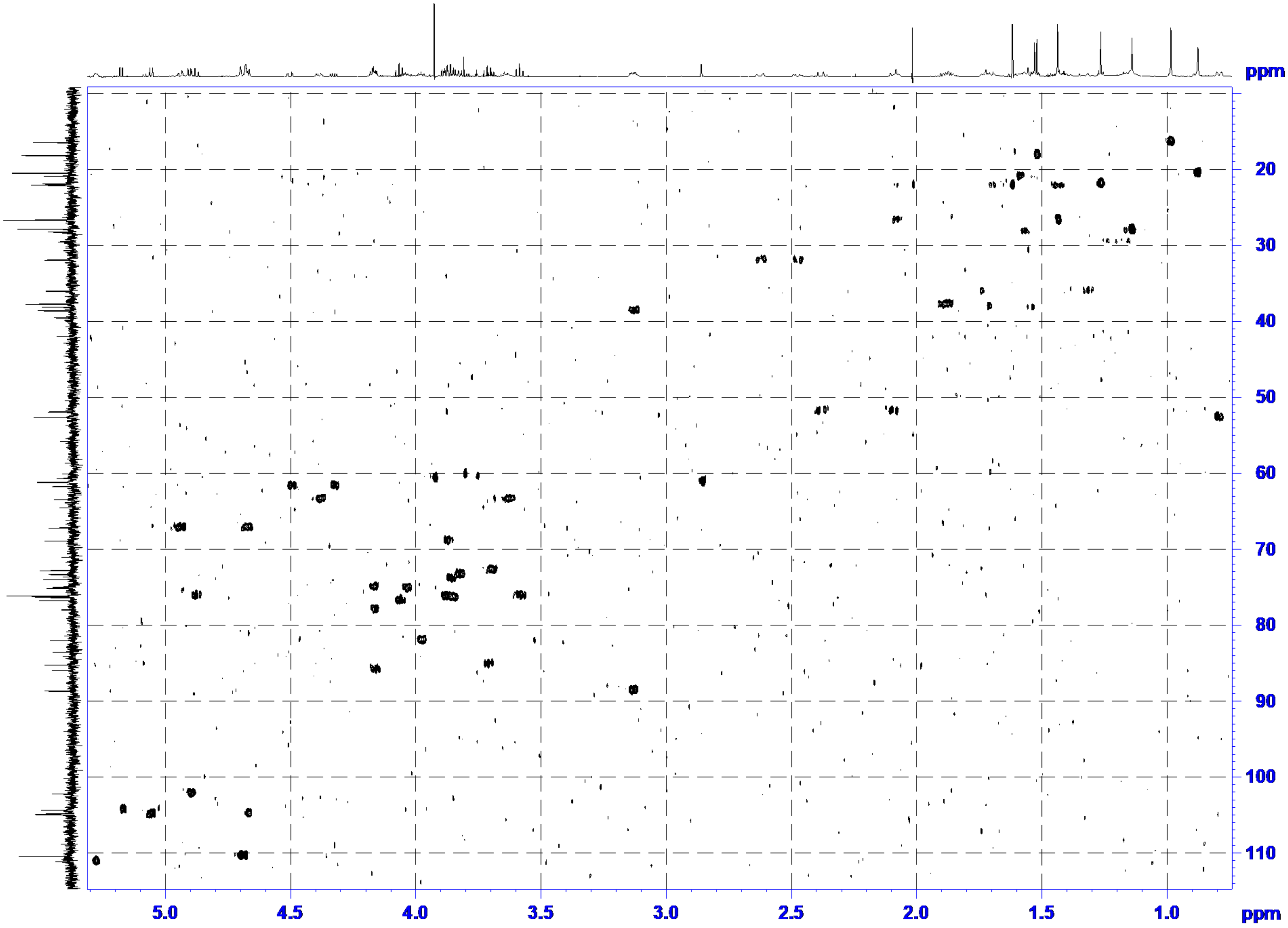

Figure S12. The HSQC (500.12 MHz) spectrum of chitonoidoside F (**2**) in C_5D_5N/D_2O (4/1).

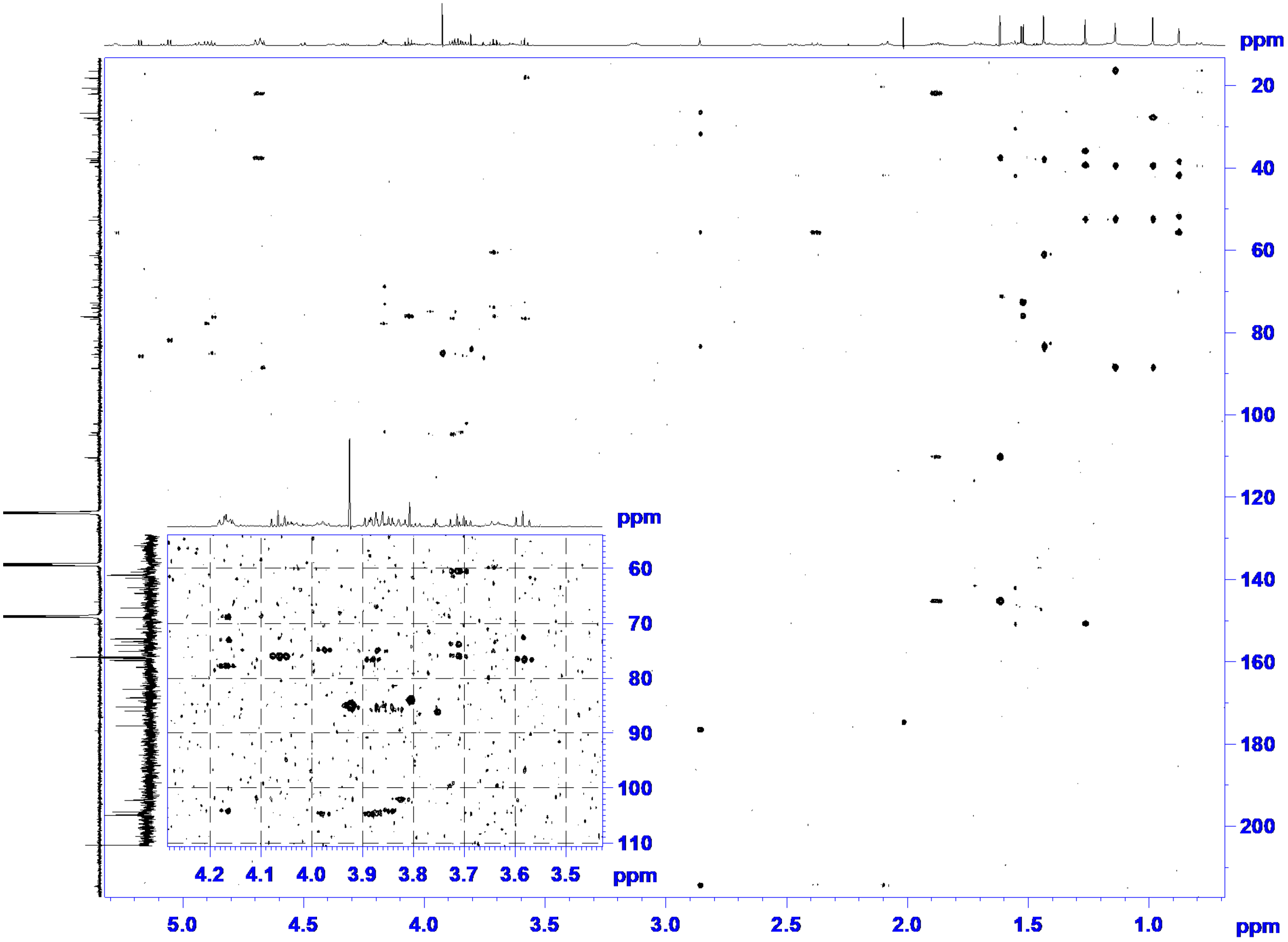

Figure S13. The HMBC (500.12 MHz) spectrum of chitonoidoside F (**2**) in C_5D_5N/D_2O (4/1).

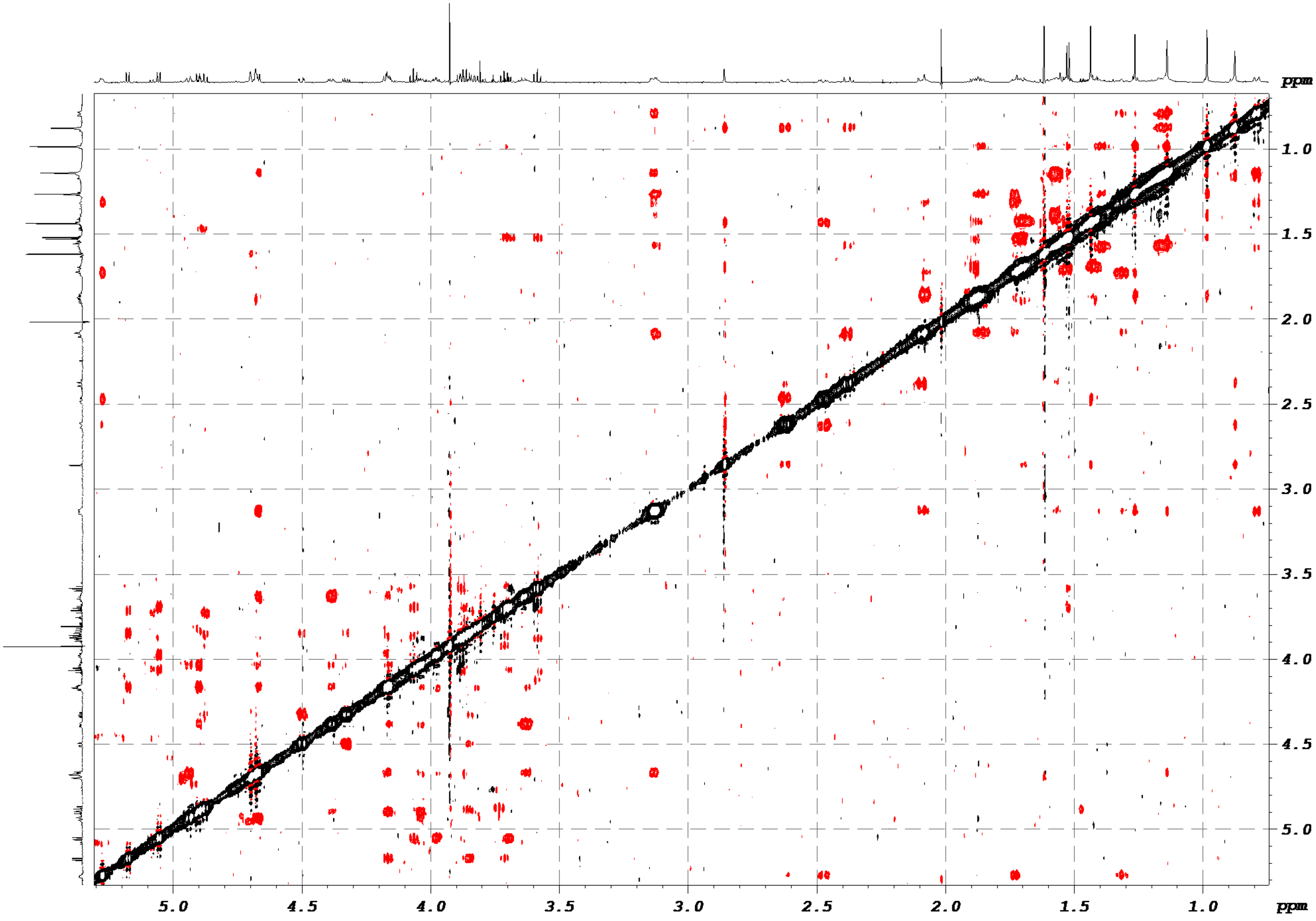

Figure S14. The ROESY (500.12 MHz) spectrum of chitonoidoside F (**2**) in C_5D_5N/D_2O (4/1).

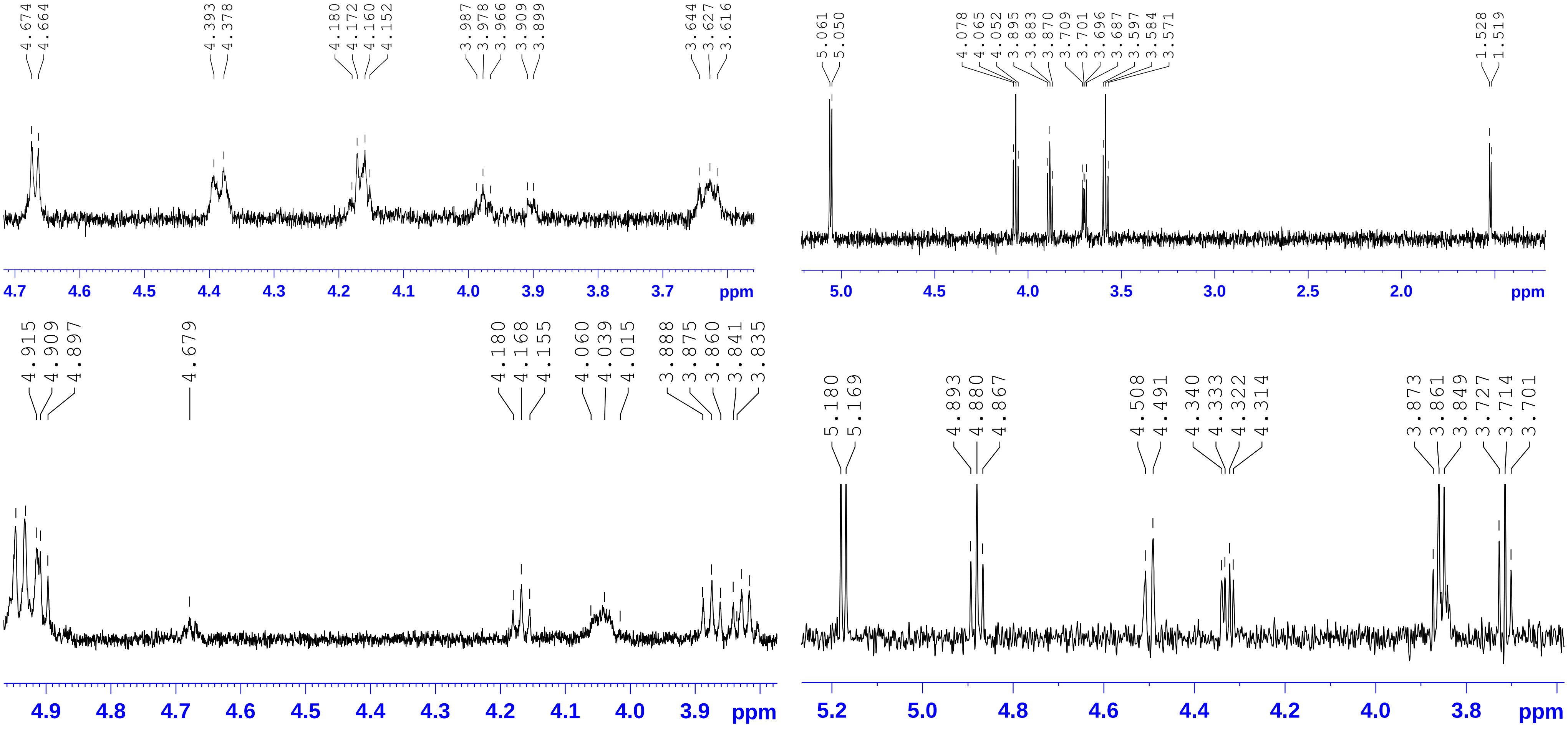

Figure S15. 1D TOCSY (500.12 MHz) spectra of Xyl1, Qui2, Glc3 and MeGlc4 of chitonoidoside F (2) in C_5D_5N/D_2O (4/1).

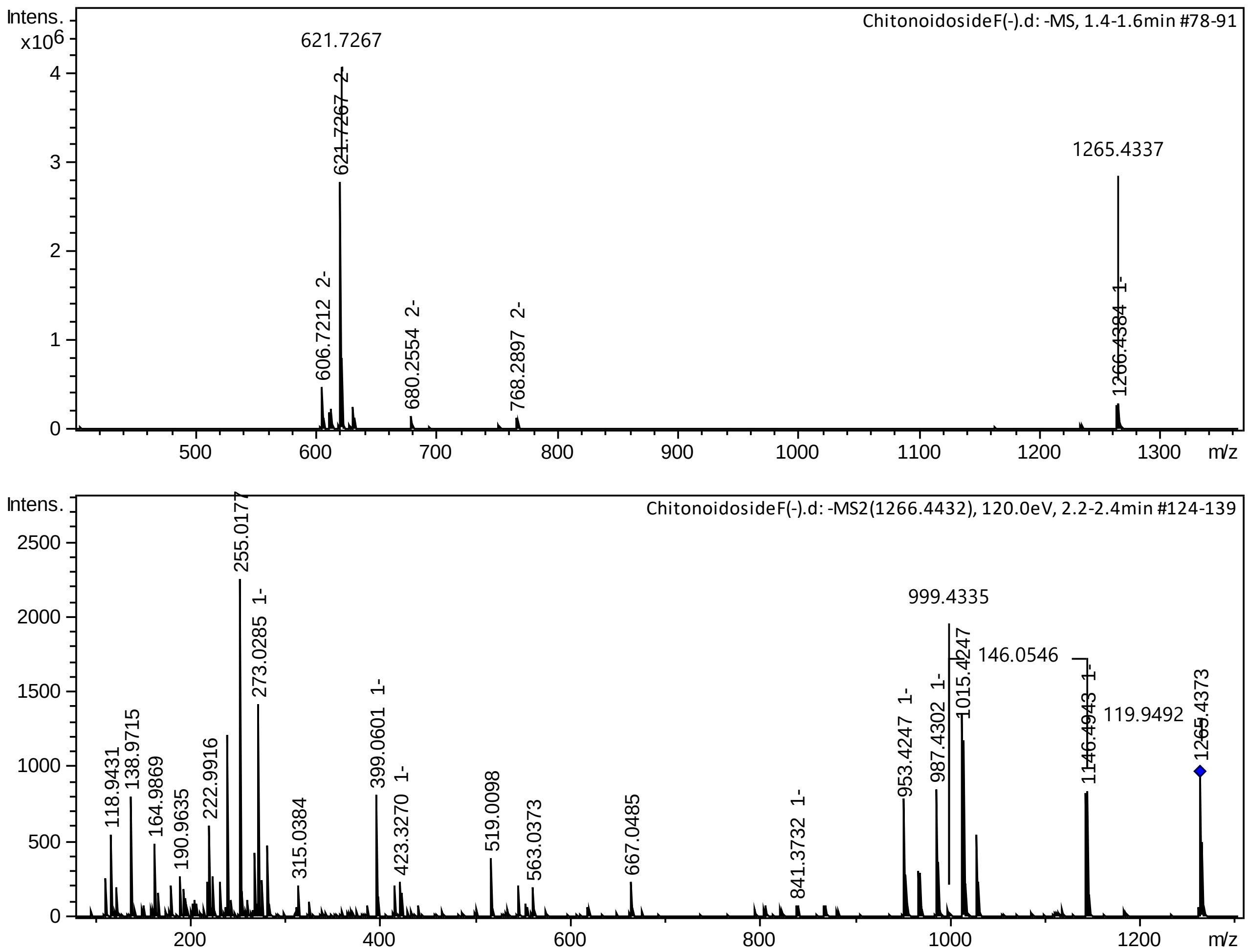

Figure S16. HR-ESI-MS and ESI-MS/MS spectra of chitonoidoside F (**2**).

Table S2. ^{13}C and ^{1}H NMR chemical shifts, HMBC and ROESY correlations of the aglycone moiety of chitonoidoside F (**2**).

Position	δ_C mult. [a]	δ_H mult. (*J* in Hz) [b]	HMBC	ROESY
1	36.0 CH_2	1.80 m		H-11, H-19
		1.32 m		H-3, H-5, H-11
2	26.7 CH_2	2.07 m		
		1.48 m		H-19, H-30
3	88.7 CH	3.13 dd (4.2; 12.0)	C: 4, 30, 31, C:1 Xyl1	H-1, H-5, H-31, H1-Xyl1
4	39.5 C			
5	52.7 CH	0.80 brd (12.2)	C: 10, 19, 30	H-1, H-3, H-31
6	20.9 CH_2	1.58 m		
7	28.3 CH_2	1.56 m		
		1.17 m		H-32
8	38.6 CH	3.13 m		H-6, H-19
9	150.9 C			
10	39.7 C			
11	111.1 CH	5.28 m	C: 10, 12, 13	H-1
12	31.9 CH_2	2.63 brd (16.9)	C: 9, 11, 13, 18	H-17, H-32
		2.48 dd (5.5; 16.9)	C: 9, 11, 13, 14, 18	
13	55.8 C			
14	42.0 C			
15	51.9 CH_2	2.38 d (15.4)	C: 13, 16, 17, 32	H-7, H-32
		2.09 d (15.4)	C: 8, 14, 16, 32	
16	214.5 C			
17	61.7 CH	2.86 s	C: 12, 13, 16, 18, 20, 21	H-21, H-32
18	176.7 C			
19	21.9 CH_3	1.27 s	C: 1, 5, 9, 10	H-8, H-30
20	83.6 C			
21	26.7 CH_3	1.44 s	C: 17, 20, 22	H-12, H-17
22	38.1 CH_2	1.71 m	C: 20, 21, 23	
		1.55 m		
23	22.0 CH_2	1.70 m		
		1.43 m		
24	37.7 CH_2	1.88 m	C: 25, 26, 27	
25	145.5 C			
26	110.4 CH_2	4.70 brs	C: 24, 27	
		4.68 brs	C: 24, 27	
27	22.1 CH_3	1.62 s	C: 24, 25, 26	
30	16.4 CH_3	0.99 s	C: 3, 4, 5, 31	H-31
31	27.9 CH_3	1.14 s	C: 3, 4, 5, 30	H-3, H-5, H-30
32	20.4 CH_3	0.88 s	C: 8, 13, 14, 15	H-7, H-12, H-17

[a]Recorded at 125.67 MHz in C_5D_5N/D_2O (4/1). [b]Recorded at 500.12 MHz in C_5D_5N/D_2O (4/1).

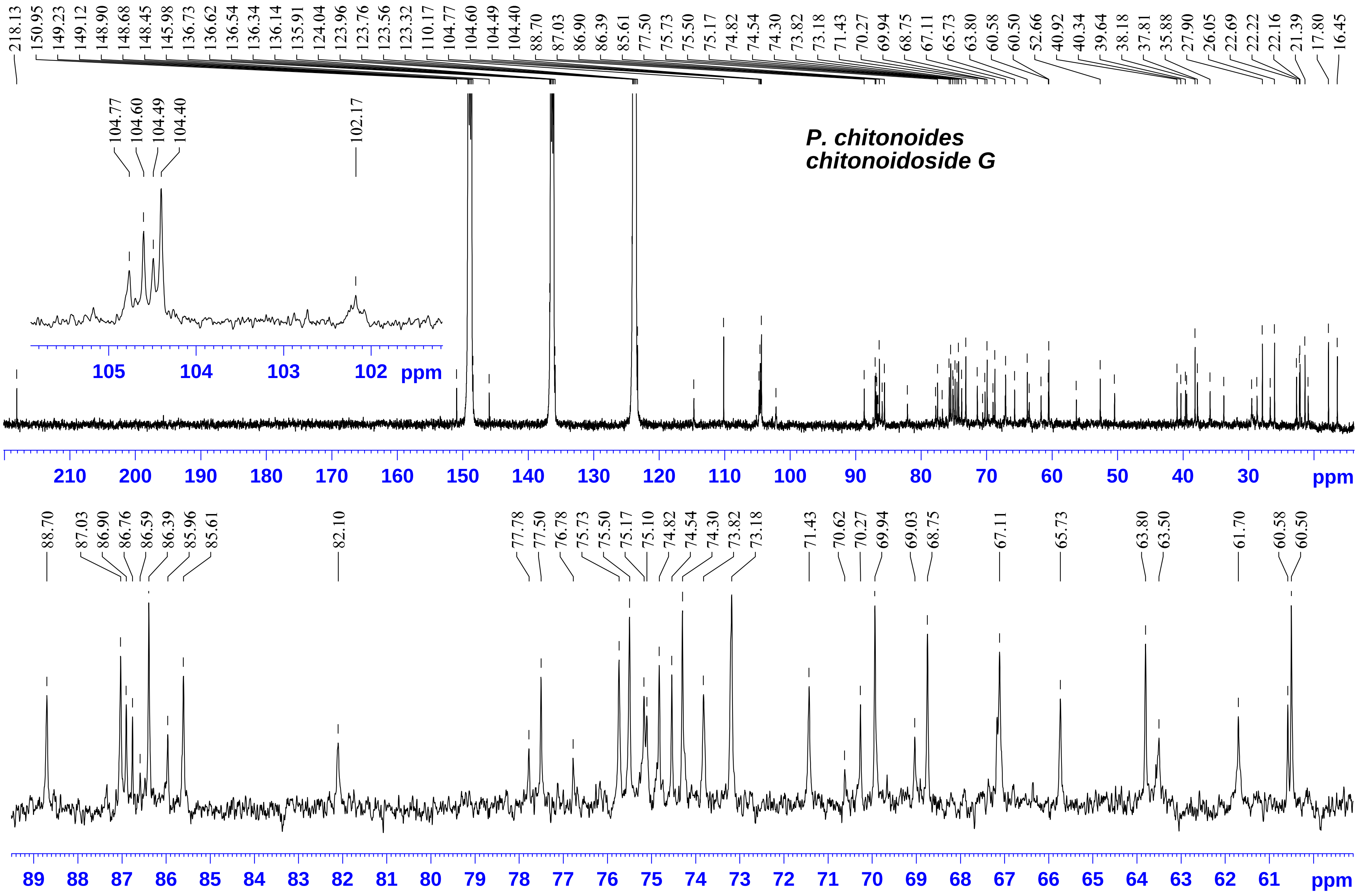

Figure S17. The ^{13}C NMR (125.67 MHz) spectrum of chitonoidoside G (**3**) in C_5D_5N/D_2O (4/1).

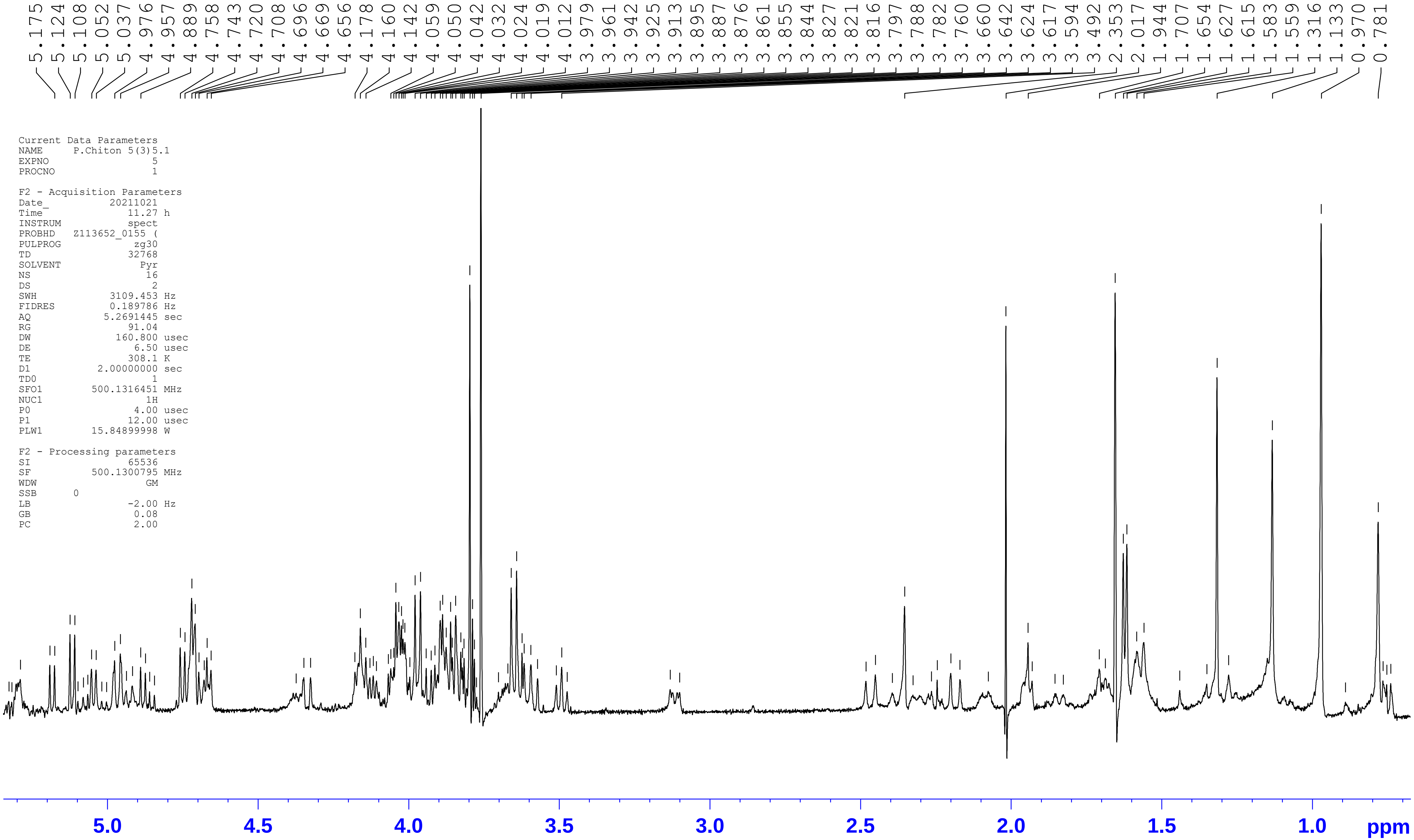

Figure S18. The ^{1}H NMR (500.12 MHz) spectrum of chitonoidoside G (**3**) in C_5D_5N/D_2O (4/1)

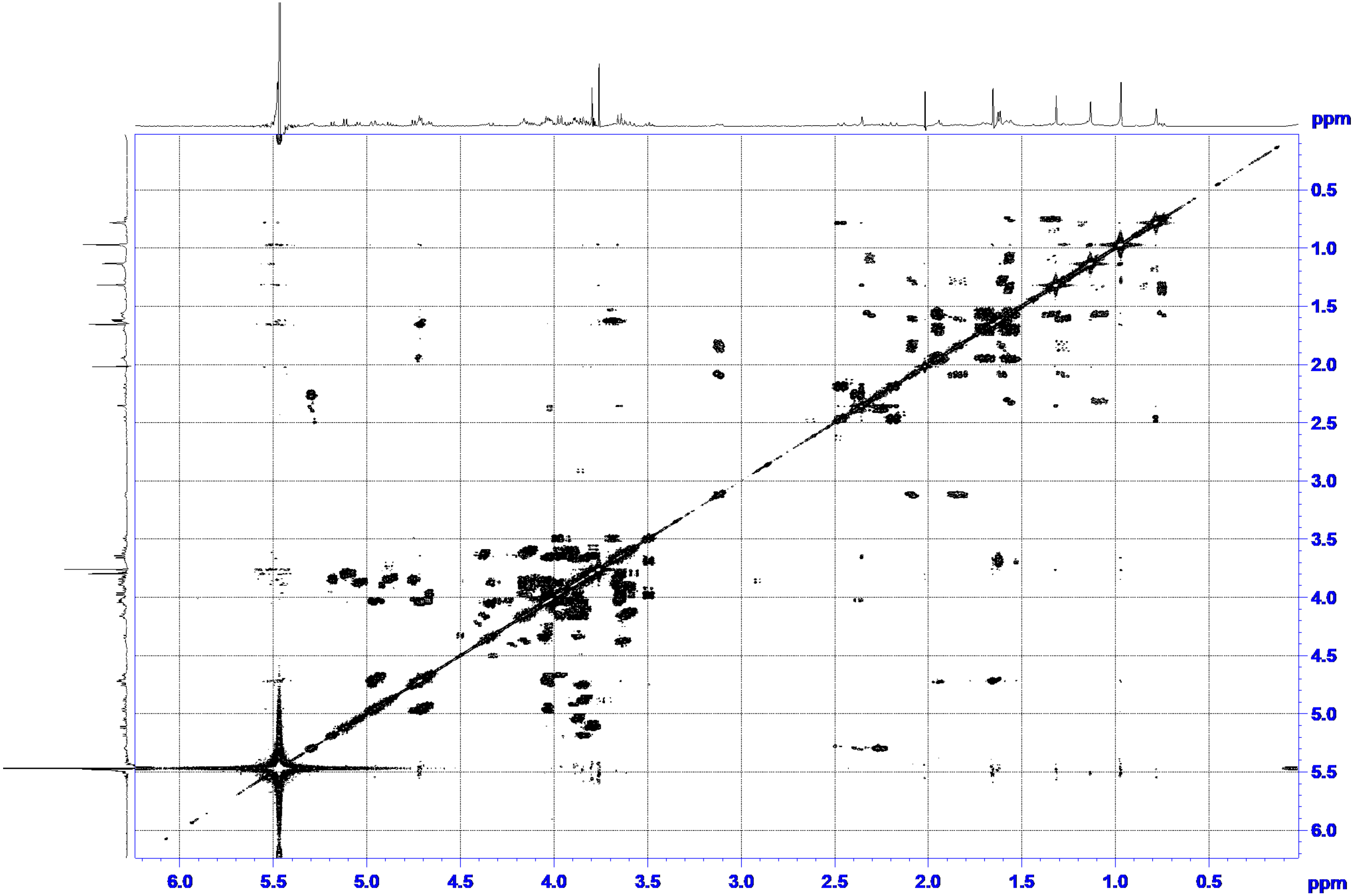

Figure S19. The COSY (500.12 MHz) spectrum of chitonoidoside G (**3**) in C_5D_5N/D_2O (4/1)

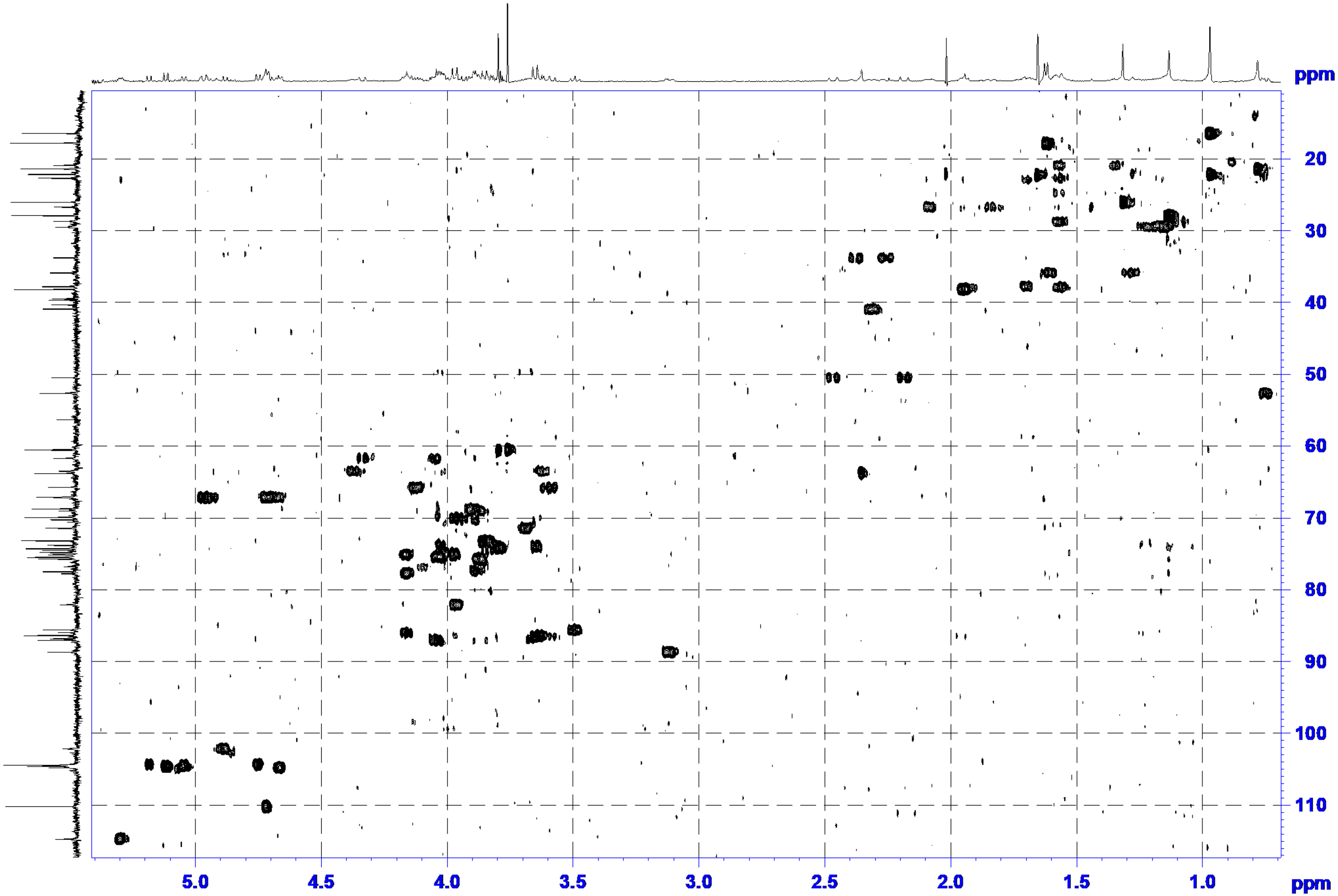

Figure S20. The HSQC (500.12 MHz) spectrum of chitonoidoside G (**3**) in C_5D_5N/D_2O (4/1)

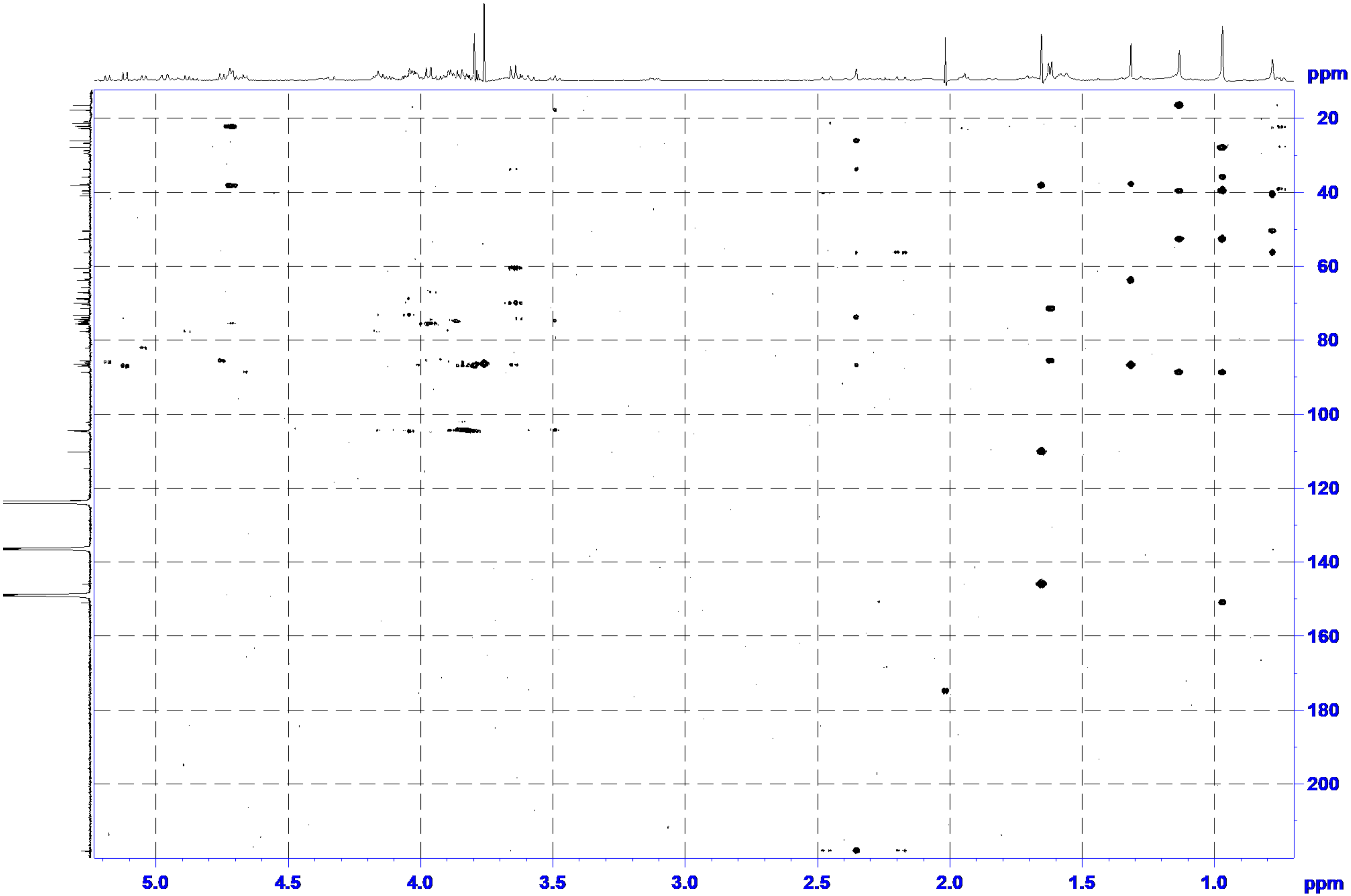

Figure S21. The HMBC (500.12 MHz) spectrum of chitonoidoside G (**3**) in C_5D_5N/D_2O (4/1)

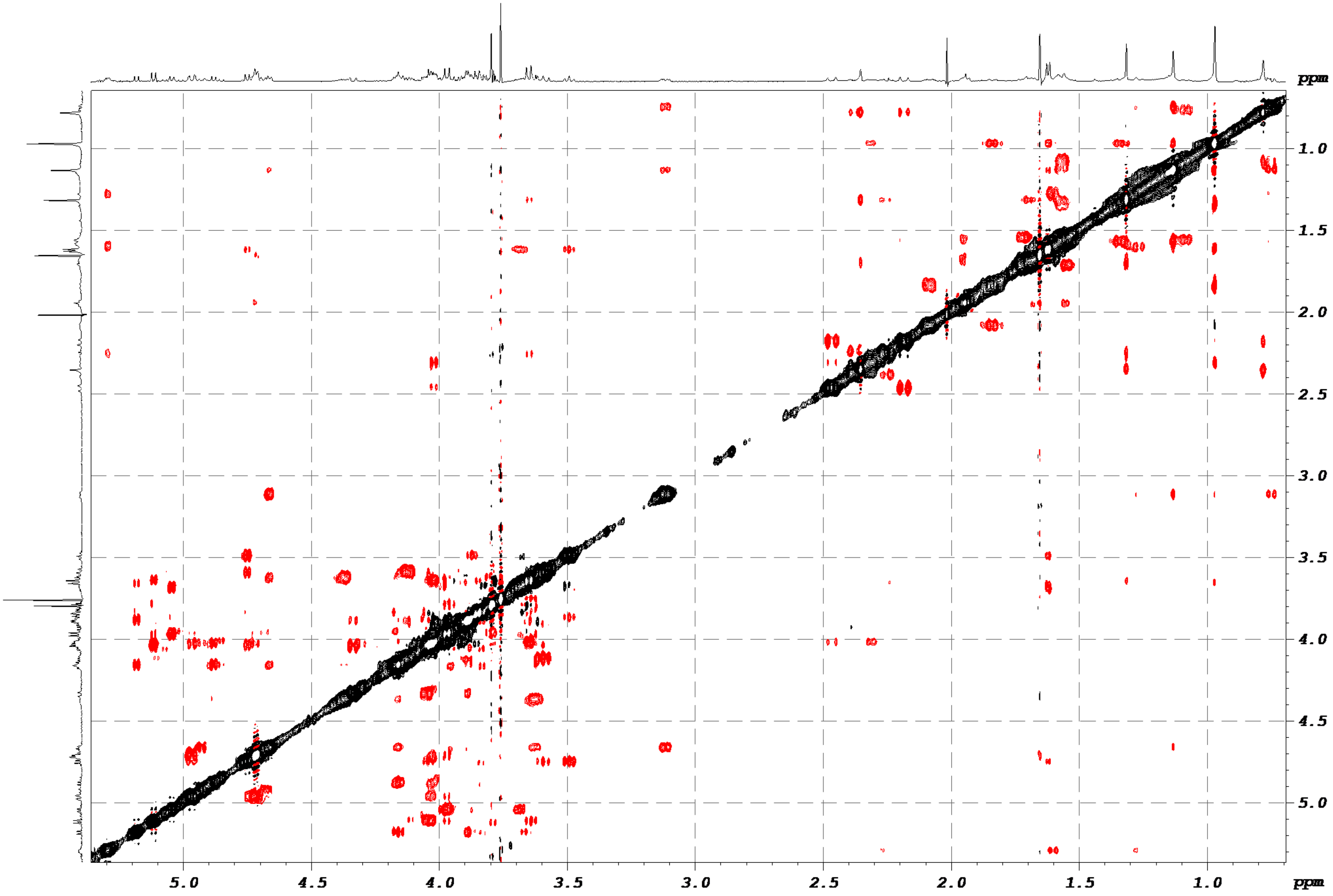

Figure S22. The ROESY (500.12 MHz) spectrum of chitonoidoside G (**3**) in C_5D_5N/D_2O (4/1)

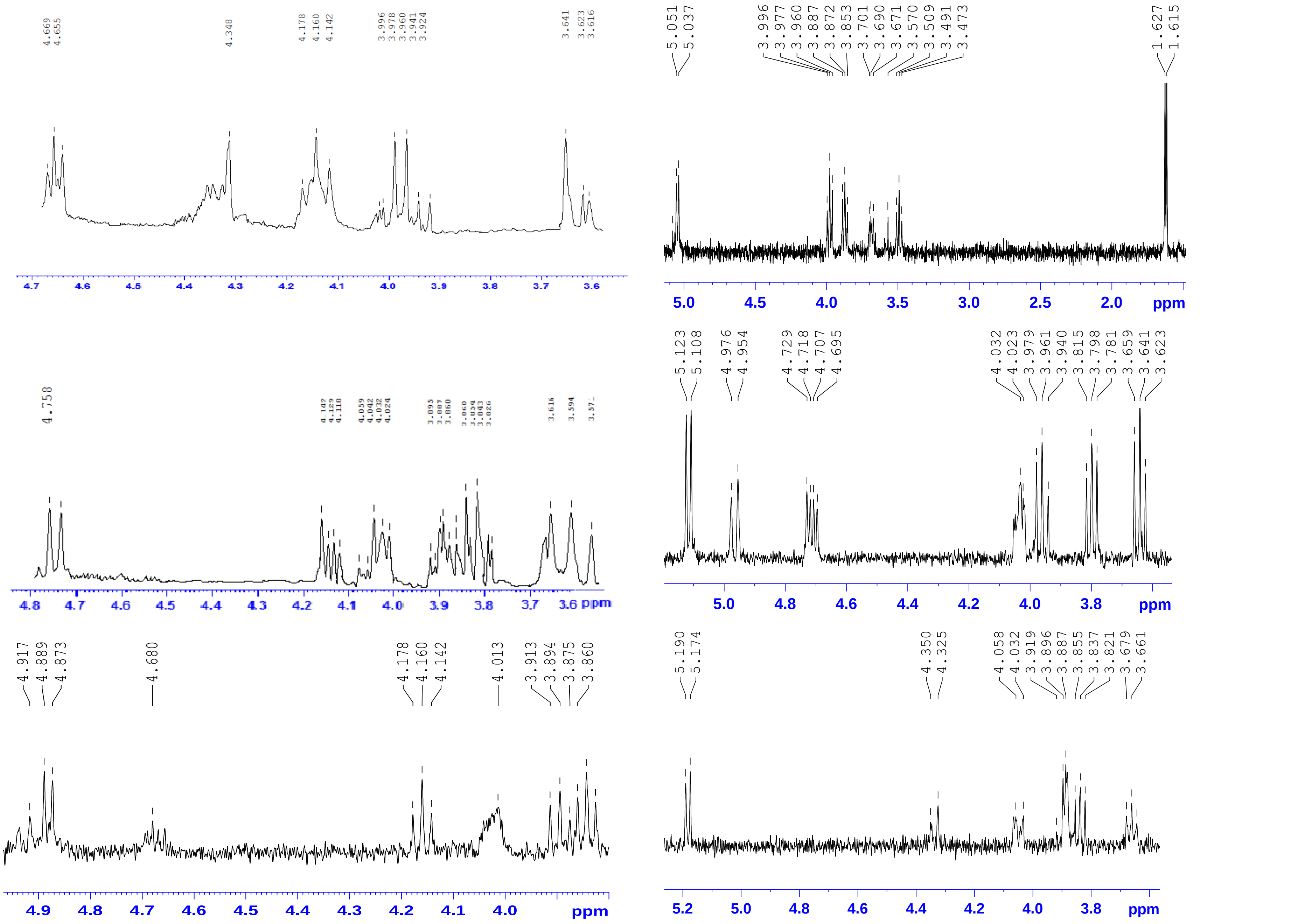

Figure S23. 1 D TOCSY (500.12 MHz) spectra of Xyl1, Qui2, Xyl3, MeGlc4, Glc5 and MeGlc6 of chitonoidoside G (**3**) in C_5D_5N/D_2O (4/1)

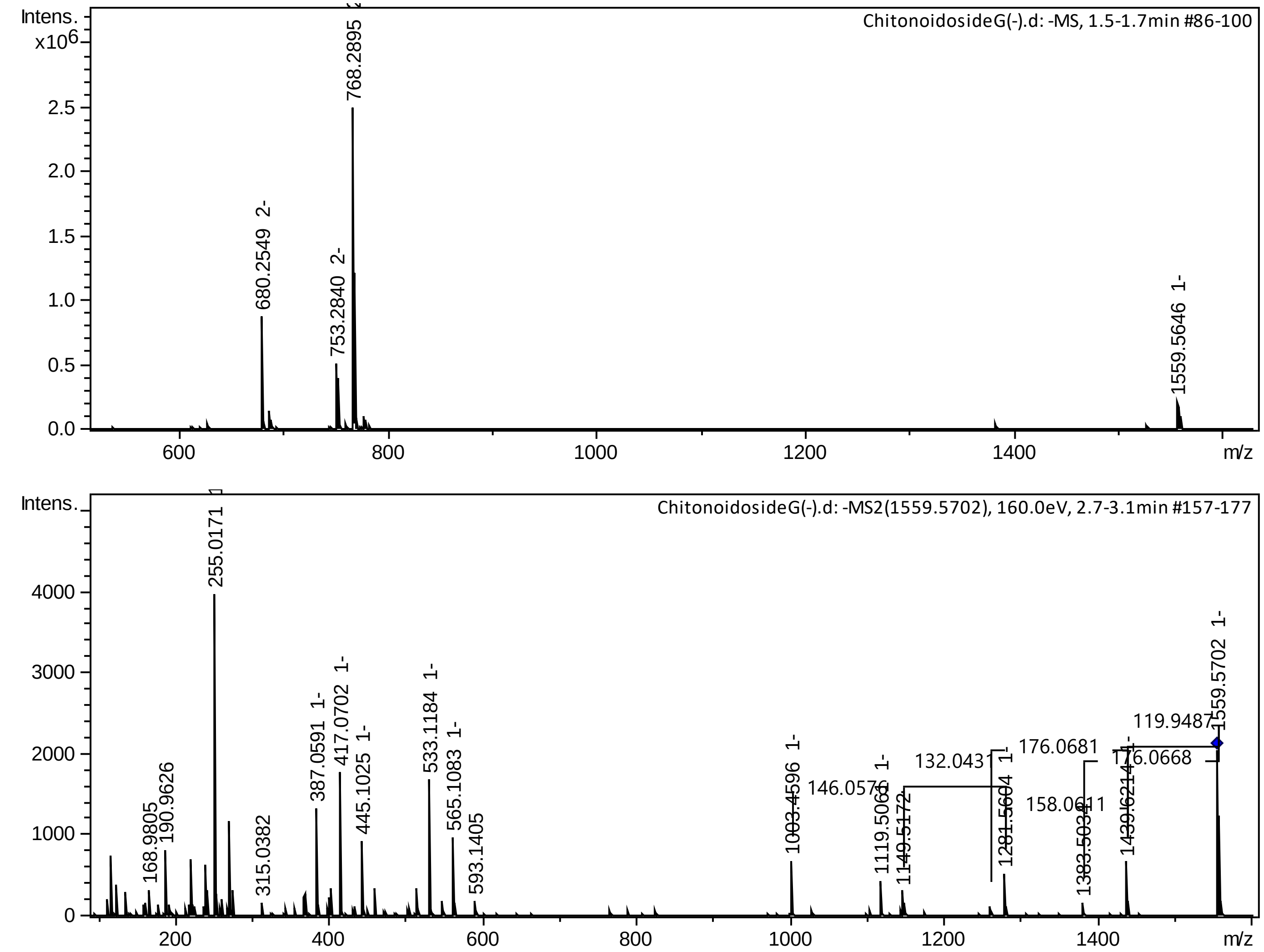

Figure S24. HR-ESI-MS and ESI-MS/MS spectra of chitonoidoside G (**3**)

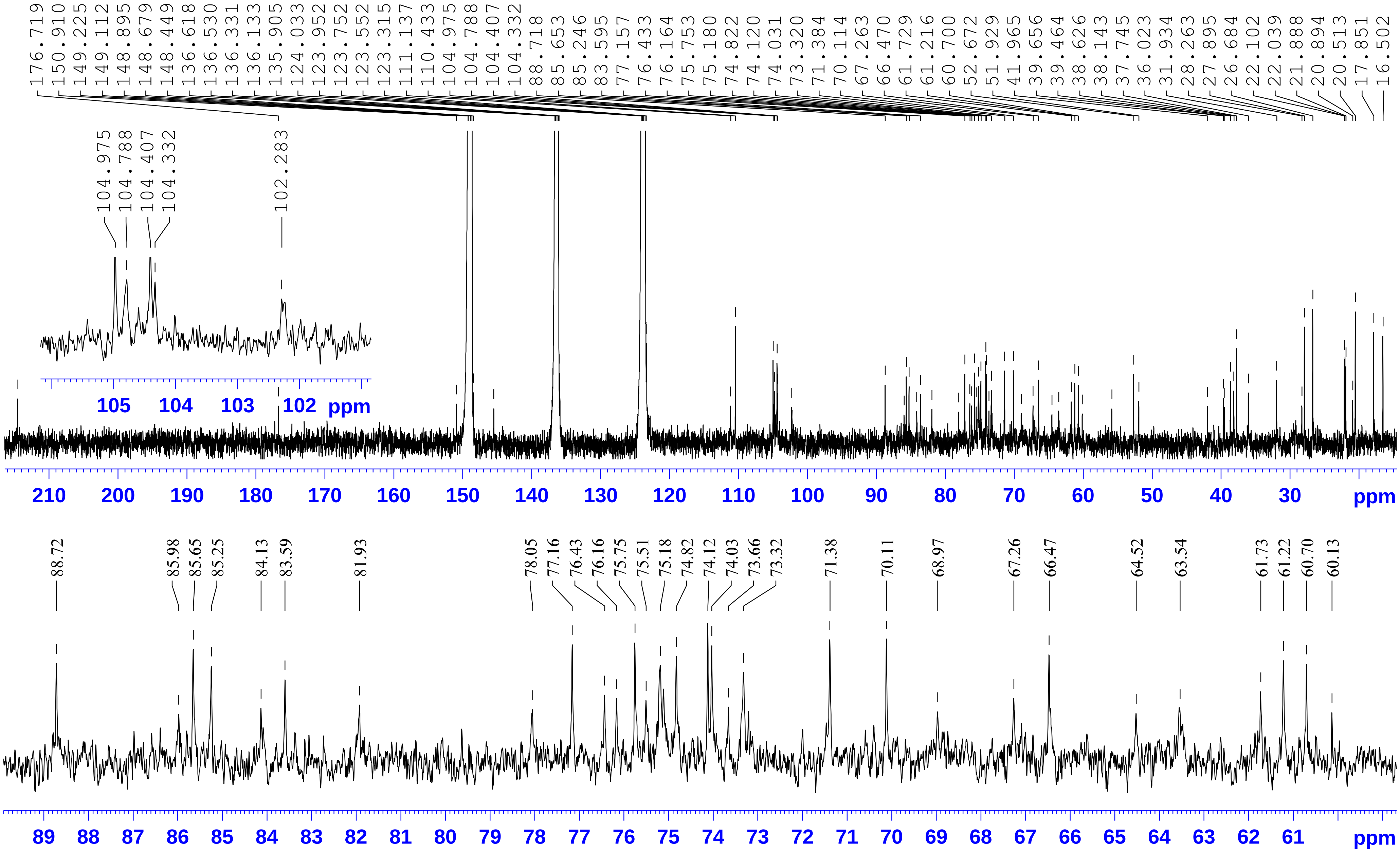

Figure S25. The ^{13}C NMR (125.67 MHz) spectrum of chitonoidoside H (**4**) in C_5D_5N/D_2O (4/1)

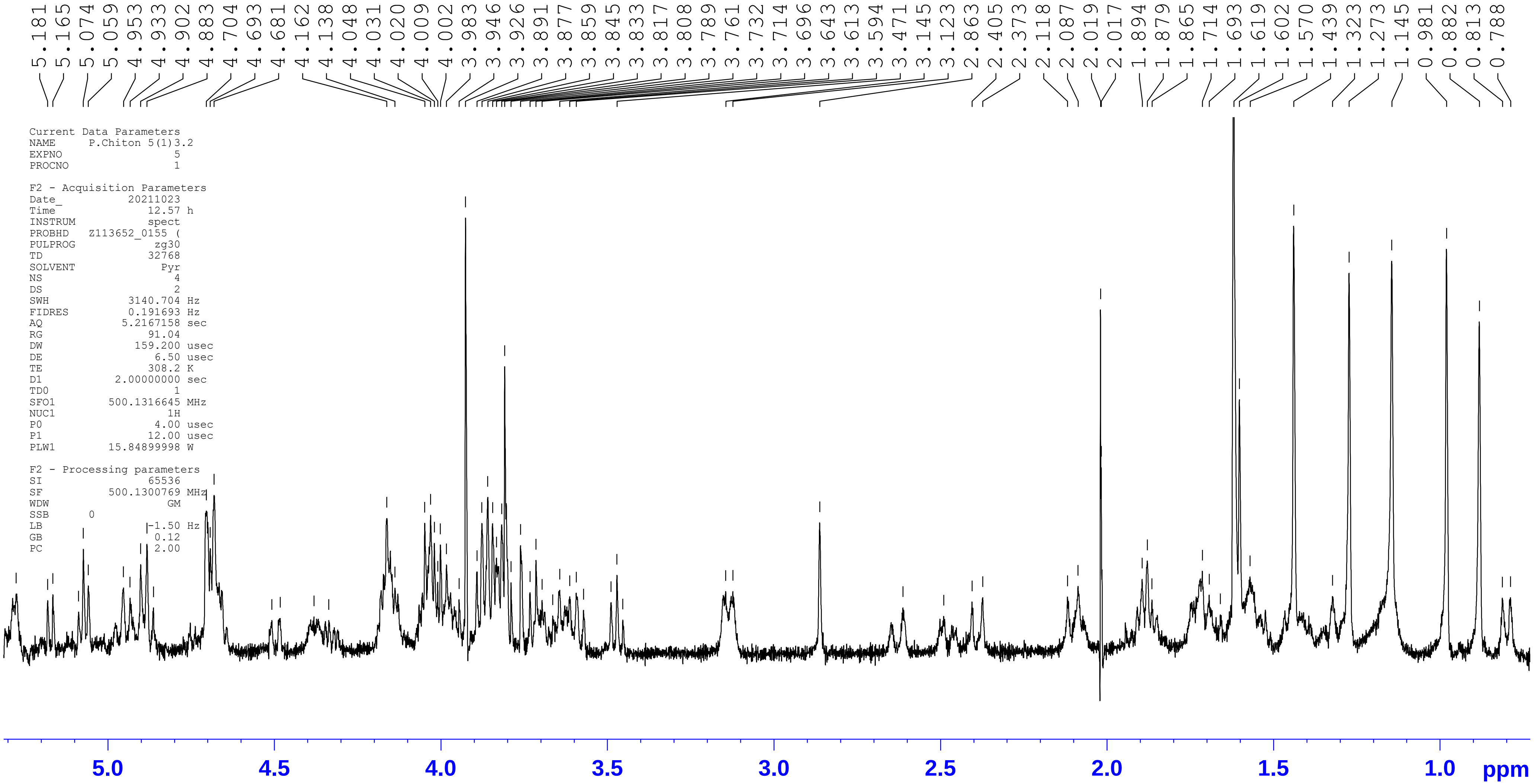

Figure S26. The ^{1}H NMR (500.12 MHz) spectrum of chitonoidoside H (**4**) in C_5D_5N/D_2O (4/1)

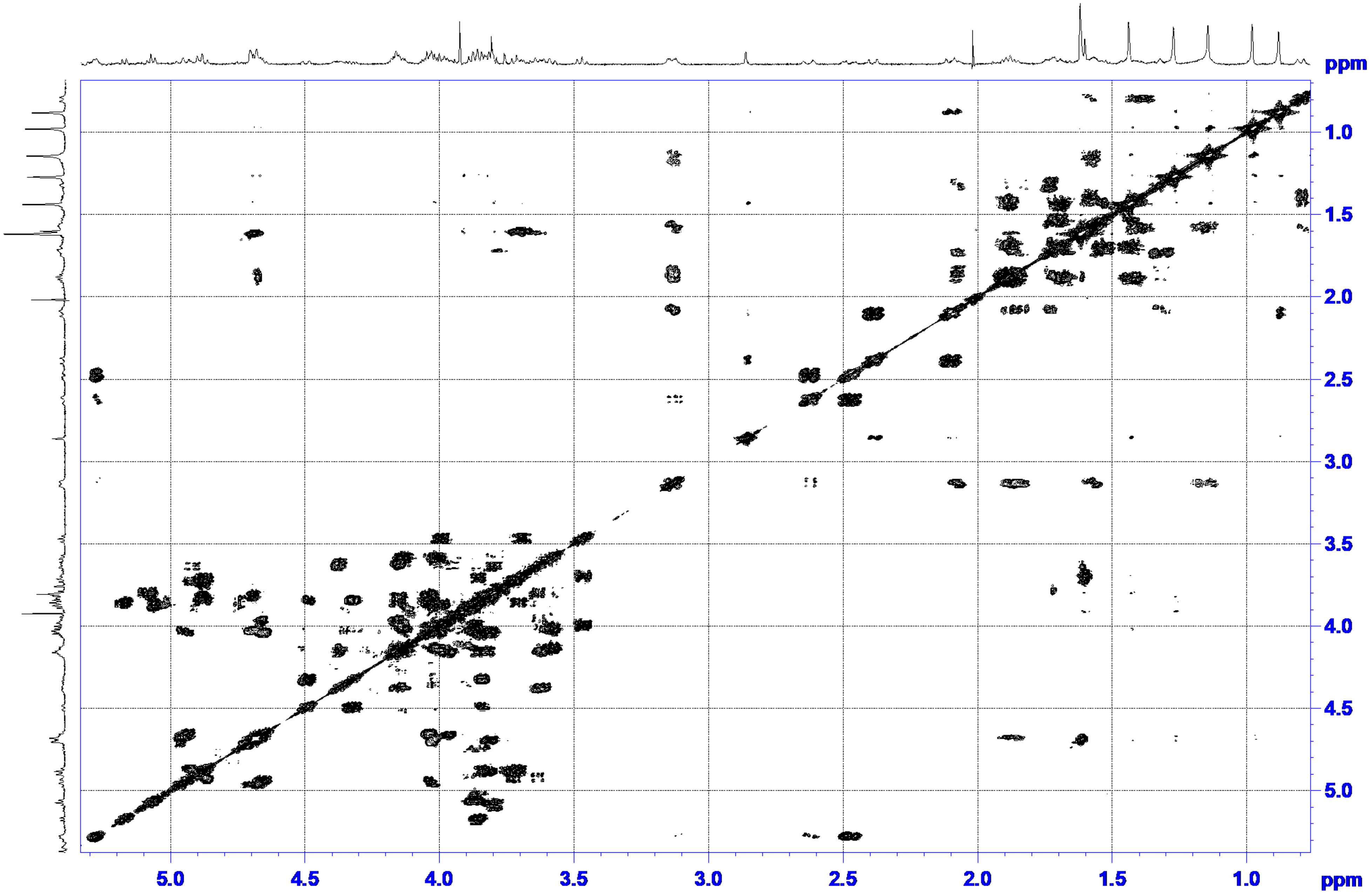

Figure S27. The COSY (500.12 MHz) spectrum of chitonoidoside H (**4**) in C_5D_5N/D_2O (4/1)

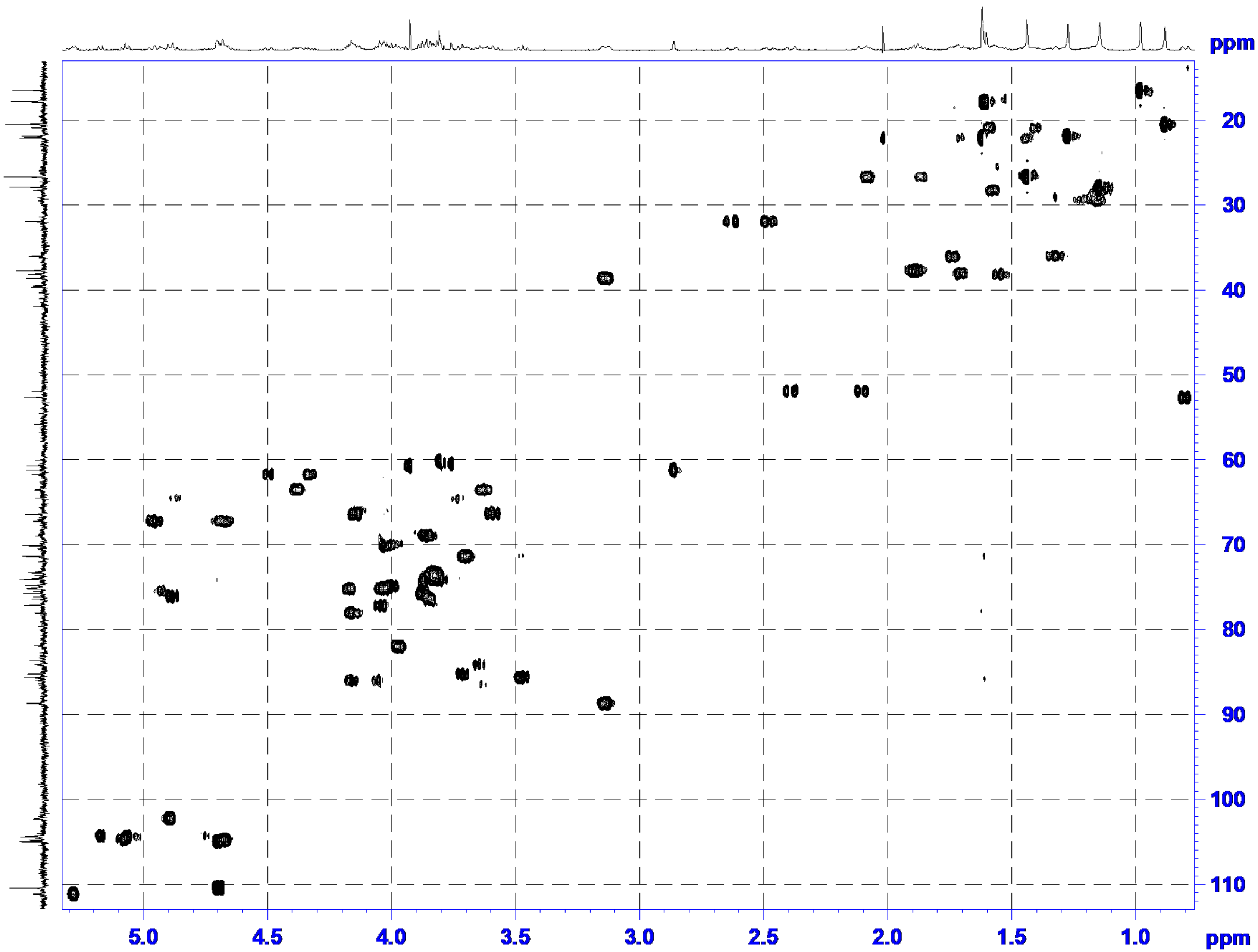

Figure S28. The HSQC (500.12 MHz) spectrum of chitonoidoside H (**4**) in C_5D_5N/D_2O (4/1)

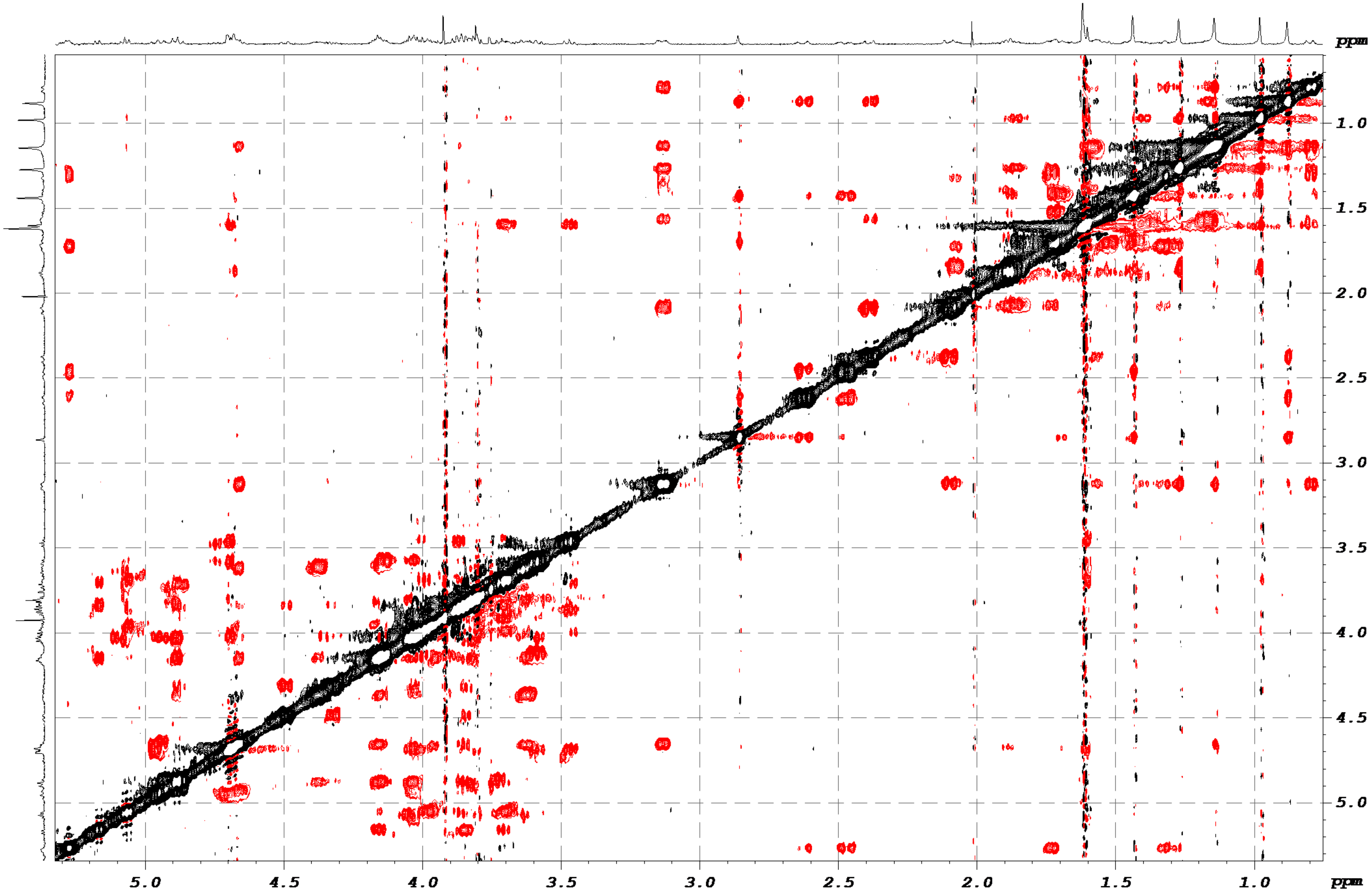

Figure S29. The ROESY (500.12 MHz) spectrum of chitonoidoside H (**4**) in C_5D_5N/D_2O (4/1)

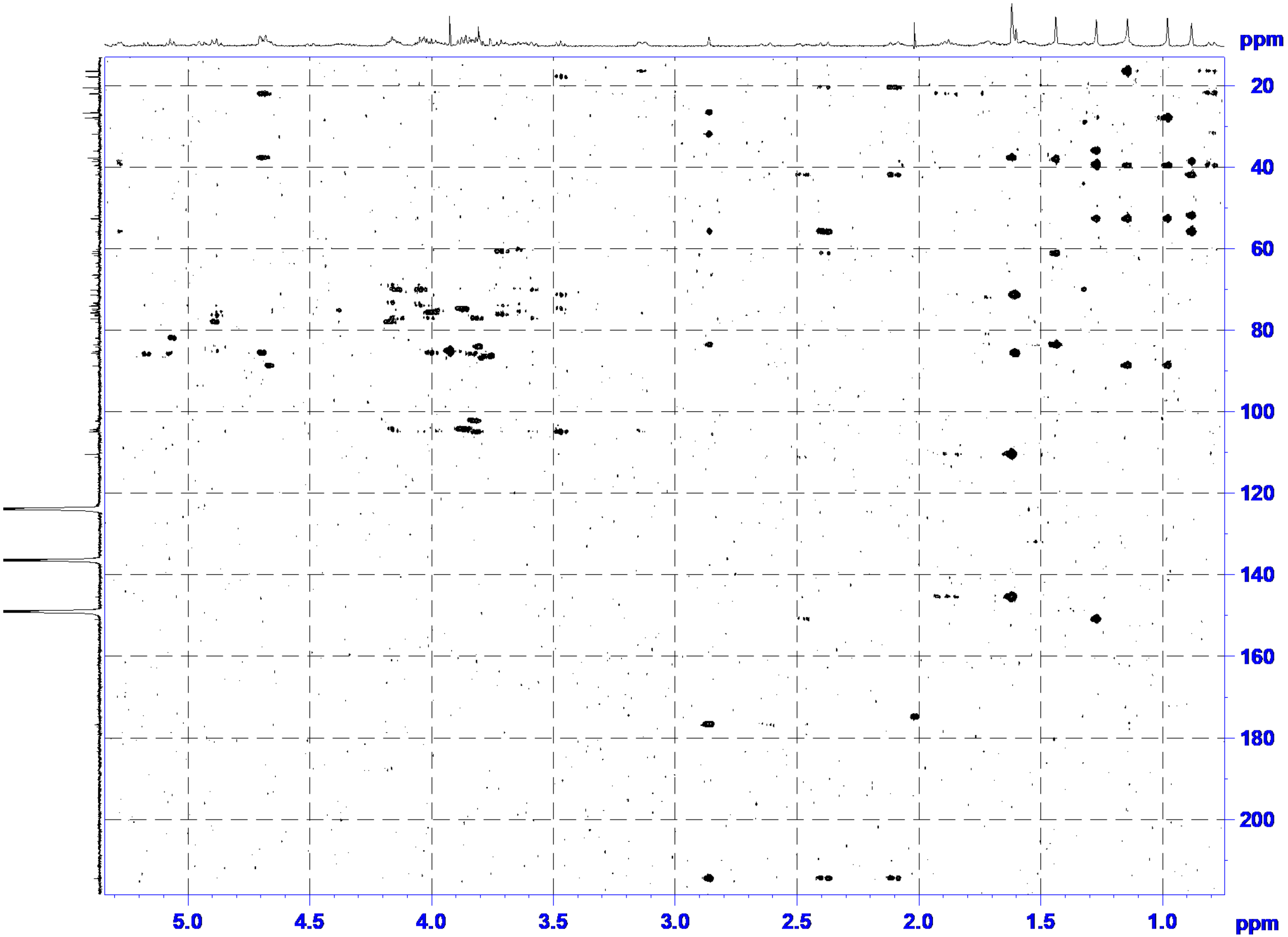

Figure S30. The HMBC (500.12 MHz) spectrum of chitonoidoside H (**4**) in C_5D_5N/D_2O (4/1)

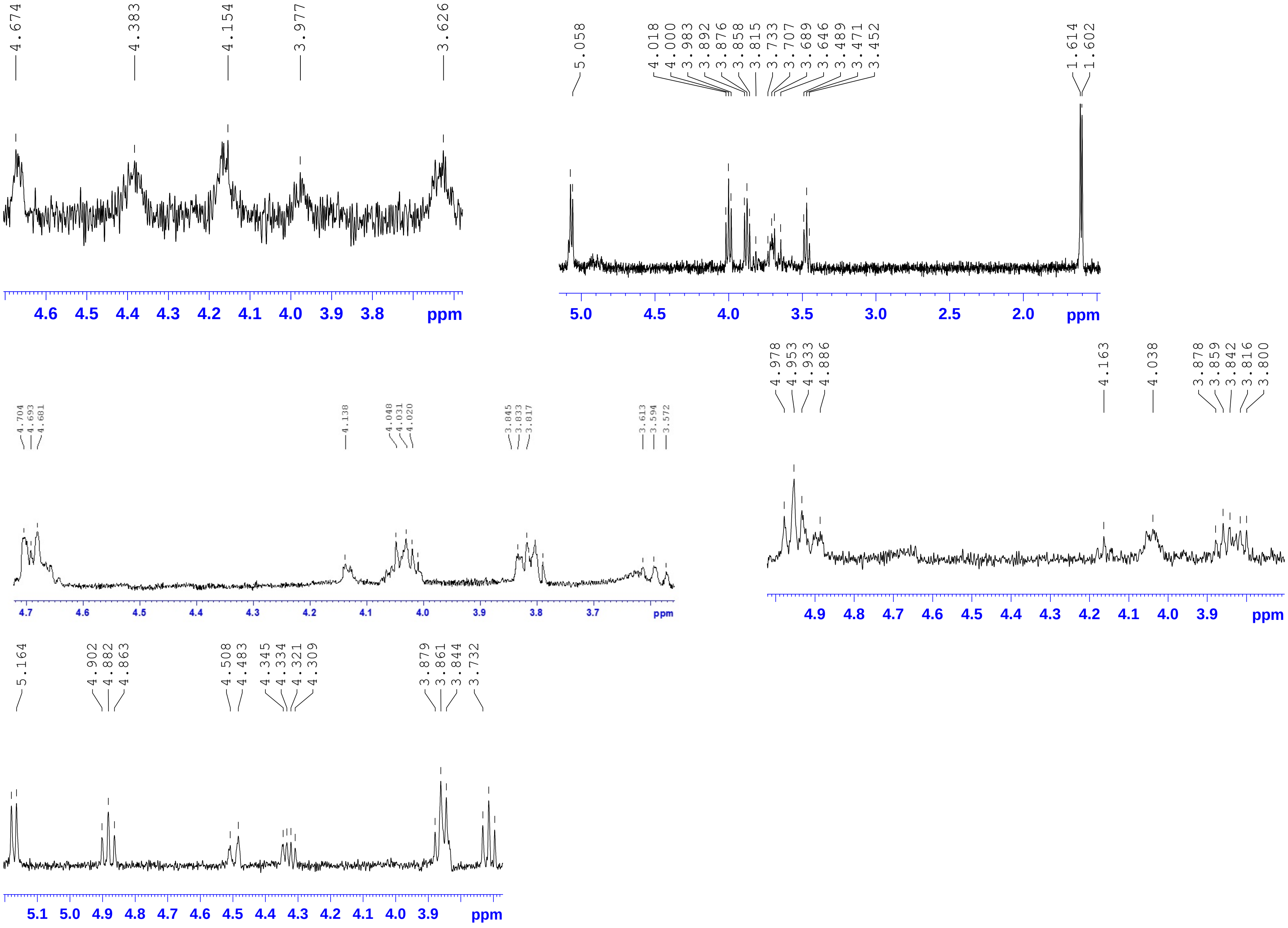

Figure S31. 1D TOCSY (500.12 MHz) spectra of Xyl1, Qui2, Xyl3, Glc4 and MeGlc5 of chitonoidoside H (**4**) in C_5D_5N/D_2O (4/1)

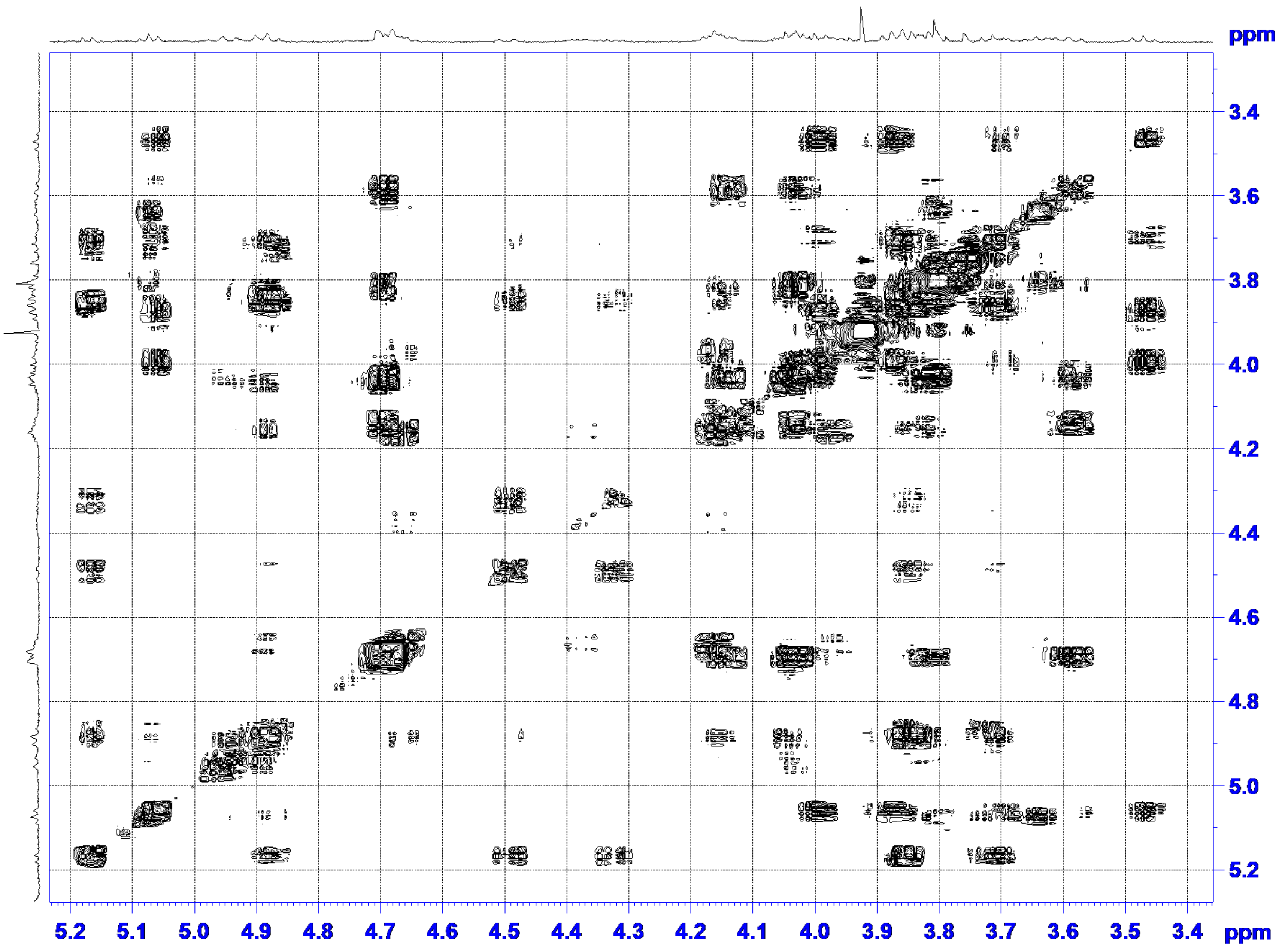

Figure S32. 2D TOCSY (500.12 MHz) spectrum of chitonoidoside H (**4**) in C_5D_5N/D_2O (4/1)

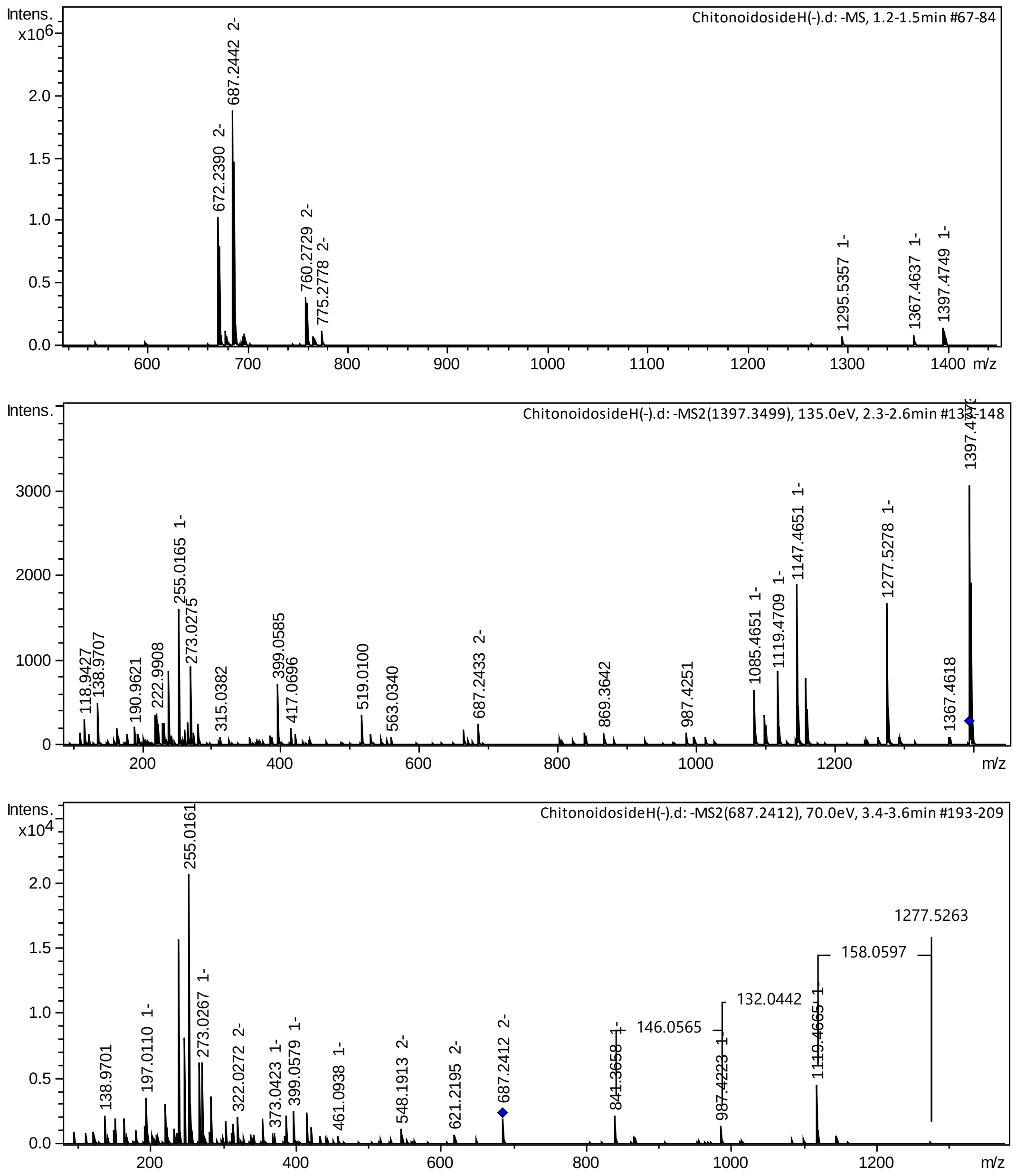

Figure S33. HR-ESI-MS and ESI-MS/MS spectra of chitonoidoside C (**4**)

Table S3. ^{13}C and ^{1}H NMR chemical shifts, HMBC and ROESY correlations of the aglycone moiety of chitonoidoside H (**4**).

Position	δ_C mult.[a]	δ_H mult. (*J* in Hz) [b]	HMBC	ROESY
1	36.0 CH_2	1.74 m		H-11
		1.32 m		H-11
2	26.7 CH_2	2.08 m		
		1.87 m		
3	88.7 CH	3.14 dd (3.8; 11.9)	C: 4, 30, 31, C:1 Xyl1	H-5, H-31, H1-Xyl1
4	39.5 C			
5	52.7 CH	0.80 brd (11.0)	C: 6, 10, 30	H-3, H-31
6	20.9 CH_2	1.57 m		H-31
		1.39 m		
7	28.3 CH_2	1.58 m		
		1.10 m		H-32
8	38.6 CH	3.13 m		H-15, H-19
9	150.9 C			
10	39.7 C			
11	111.1 CH	5.28 m	C: 8, 13	H-1
12	31.9 CH_2	2.63 d (17.3)	C: 9, 13, 18	H-17, H-32
		2.48 dd (5.5; 17.3)	C: 9, 11, 13, 14, 18	H-21
13	55.8 C			
14	42.0 C			
15	51.9 CH_2	2.39 d (15.7)	C: 13, 16, 17, 32	H-32
		2.10 d (15.7)	C: 8, 14, 16, 32	H-8
16	214.5 C			
17	61.2 CH	2.87 s	C: 12, 13, 16, 18, 20, 21	H-12, H-21, H-22, H-32
18	176.7 C			
19	21.9 CH_3	1.27 s	C: 1, 5, 9, 10	H-1, H-2, H-8, H-30
20	83.6 C			
21	26.7 CH_3	1.44 s	C: 17, 20, 22	H-12, H-17
22	38.1 CH_2	1.70 m	C: 20, 21, 23	
		1.54 m	C: 20, 21	
23	22.1 CH_2	1.70 m		
		1.44 m		
24	37.7 CH_2	1.89 m	C: 23, 25, 26, 27	
25	145.5 C			
26	110.4 CH_2	4.71 brs	C: 24, 27	
		4.68 brs	C: 24, 27	
27	22.0 CH_3	1.62 s	C: 24, 25, 26	
30	16.5 CH_3	0.98 s	C: 3, 4, 5, 31	H-31
31	27.9 CH_3	1.15 s	C: 3, 4, 5, 30	H-3, H-5, H-30
32	20.5 CH_3	0.88 s	C: 8, 13, 14, 15	H-7, H-12, H-17

[a]Recorded at 125.67 MHz in C_5D_5N/D_2O (4/1). [b]Recorded at 500.12 MHz in C_5D_5N/D_2O (4/1).
